# The double round-robin population unravels the genetic architecture of grain size in barley

**DOI:** 10.1093/jxb/erac369

**Published:** 2022-09-12

**Authors:** Asis Shrestha, Francesco Cosenza, Delphine van Inghelandt, Po-Ya Wu, Jinquan Li, Federico A Casale, Marius Weisweiler, Benjamin Stich

**Affiliations:** Institute for Quantitative Genetics and Genomics of Plants, Biology Department, Heinrich Heine University, Dusseldorf, Germany; Institute for Quantitative Genetics and Genomics of Plants, Biology Department, Heinrich Heine University, Dusseldorf, Germany; Institute for Quantitative Genetics and Genomics of Plants, Biology Department, Heinrich Heine University, Dusseldorf, Germany; Institute for Quantitative Genetics and Genomics of Plants, Biology Department, Heinrich Heine University, Dusseldorf, Germany; Max Planck Institute for Plant Breeding Research, Cologne, Germany; Institute for Quantitative Genetics and Genomics of Plants, Biology Department, Heinrich Heine University, Dusseldorf, Germany; Institute for Quantitative Genetics and Genomics of Plants, Biology Department, Heinrich Heine University, Dusseldorf, Germany; Institute for Quantitative Genetics and Genomics of Plants, Biology Department, Heinrich Heine University, Dusseldorf, Germany; Max Planck Institute for Plant Breeding Research, Cologne, Germany; Cluster of Excellence on Plant Sciences, From Complex Traits towards Synthetic Modules, Heinrich Heine University, Dusseldorf, Germany; CIMMYT, Mexico

**Keywords:** Barley, grain size, grain weight, multi-parent population, quantitative trait locus (QTL), yield-related traits

## Abstract

Grain number, size and weight primarily determine the yield of barley. Although the genes regulating grain number are well studied in barley, the genetic loci and the causal gene for sink capacity are poorly understood. Therefore, the primary objective of our work was to dissect the genetic architecture of grain size and weight in barley. We used a multi-parent population developed from a genetic cross between 23 diverse barley inbreds in a double round-robin design. Seed size-related parameters such as grain length, grain width, grain area and thousand-grain weight were evaluated in the HvDRR population comprising 45 recombinant inbred line sub-populations. We found significant genotypic variation for all seed size characteristics, and observed 84% or higher heritability across four environments. The quantitative trait locus (QTL) detection results indicate that the genetic architecture of grain size is more complex than previously reported. In addition, both cultivars and landraces contributed positive alleles at grain size QTLs. Candidate genes identified using genome-wide variant calling data for all parental inbred lines indicated overlapping and potential novel regulators of grain size in cereals. Furthermore, our results indicated that sink capacity was the primary determinant of grain weight in barley.

## Teaser

Multi-parent population has uncovered a natural allelic series across quantitative trait loci associated with grain size and weight that will contribute to identifying causal genes and yield improvement in barley.

## Introduction

Barley (*Hordeum vulgare* L.) is one of the economically most important cereal crops after rice, wheat, and maize (Newton *et al.*, 2011). Barley grain is primarily used in the animal feed and brewing industries (Dawson *et al.*, 2015). Large and plump grains contain a higher proportion of extractable sugar compared with small grains, and thus, are ideal for brewing (Walker *et al.*, 2013). Identifying the genetic regulators of natural grain size variation in barley is essential to creating additional phenotypic diversity by transgenesis (Scheben and Edwards, 2018). However, classical breeding is also facilitated by such information when used for marker-assisted selection.

Grain development is a complex process governed by multiple genes (Li *et al.*, 2018). The genetic basis of different grain size parameters has been extensively studied in rice compared with other cereals. Several quantitative trait loci (QTLs) and the underlying genes controlling grain length (GL), grain width (GW) and weight have been isolated using map-based cloning in rice (Li *et al.*, 2019). Most of the genes control cell proliferation and cell expansion of the hulls (Li *et al.*, 2018). The described genes in rice are primarily transcriptional regulators or signalling proteins involved in G-protein, MAP kinase, and hormonal signalling pathways (Li *et al.*, 2018).

A considerable effort has also been made to dissect the genetic architecture of kernel size in maize. For example, association and linkage mapping studies have identified genetic loci linked to grain size and weight in maize (Wang *et al.*, 2013; Liu *et al*., 2014, 2017b; Chen *et al.*, 2016; Zhang *et al.*, 2017; Hao *et al.*, 2019; Li *et al.*, 2021). Similarly, comparative genomics and transgenic approaches were used to identify and validate the functional wheat orthologs of rice (Bednarek *et al.*, 2012; Ma *et al.*, 2016; Sajjad *et al.*, 2017) and Arabidopsis genes known to control seed size (Ma *et al.*, 2015; Liu *et al.*, 2020). Likewise, association mapping and linkage mapping studies, as well as functional validation of underlying genes, were performed in wheat (Kato *et al.*, 2000; Gegas *et al.*, 2010; Bennett *et al.*, 2012; Zhang *et al.*, 2012; Hu *et al.*, 2016; Simmonds *et al.*, 2016; Geng *et al.*, 2017; Li and Yang, 2017).

In contrast to other cereals, the genetic regulation of grain size is not well studied in barley. The genes controlling spikelet branching influence the average seed weight in barley (Zwirek *et al.*, 2019). Until now, five genes associated with spikelet branching, i.e. controlling two versus six-rowed spikes, were characterized in barley (Komatsuda *et al.*, 2007; Ramsay *et al.*, 2011; Koppolu *et al.*, 2013; Bull *et al.*, 2017; Youssef *et al.*, 2017). However, natural allelic variations were only reported for *vulgare six-rowed spike 1* (*vrs1*)and *vrs5/int-c* (Casas *et al.*, 2018).

Only a few mapping studies were performed to identify the genetic factors controlling grain size-related characters in barley. Among them, some studies used bi-parental populations to identify genomic loci associated with grain size characters such as GL, GW, grain area (GA) and thousand-grain weight (TGW; Walker *et al.*, 2013; Zhou *et al.*, 2016; Wang *et al*., 2019, 2021; Watt *et al*., 2019, 2020). Several common QTLs for grain size characters, such as GL, TGW, GW, GW/GL ratio, and GA, were identified in a European barley cultivar association panel (Xu *et al.*, 2018).

Genetic mapping in a bi-parental population only uses a small fraction of species-wide allelic diversity, and the limited number of recombination events within the population leads to a low resolution of mapping (Scott *et al.*, 2020). An alternative approach is to use genome-wide association studies (GWAS), which improves the localization of QTLs. Nevertheless, the GWAS approach has limitations, including false marker-trait associations occurring due to confounding effects of population structure and lack of power to detect the minor alleles in the population (Stich *et al*., 2006, 2008). Therefore, multi-parent populations (MPPs) are used by plant geneticists and take advantage of linkage and association mapping (Stich, 2009). Nested association mapping (NAM) is a widely used MPP and developed by crossing a diverse set of inbreds to a common founder line, producing multiple recombinant inbred line (RIL) families of bi-parental crosses (Yu *et al.*, 2008; Buckler *et al.*, 2009; Garin *et al.*, 2021). Stich (2009) and Klasen *et al.* (2012) investigated alternative crossing designs to create MPP populations concerning the power of QTL detection. The former computer simulation study found that a double round-robin crossing scheme to develop the MPP might be a resource-effective approach for the number of required crosses, without compromising the power of QTL detection, compared with half diallel crossing schemes.

The objectives of this study are (i) to demonstrate the utility of the new MPP, the double round-robin population of barley (HvDRR) by exemplarily dissecting the genetic architecture of grain size; (ii) to estimate the allelic series underlying each QTL; (iii) to identify candidate genes for the detected QTLs; and (iv) to identify promising QTLs and sub-populations for fine mapping and QTL cloning.

## Materials and methods

### Plant materials and genotyping

**Fig. 1.**
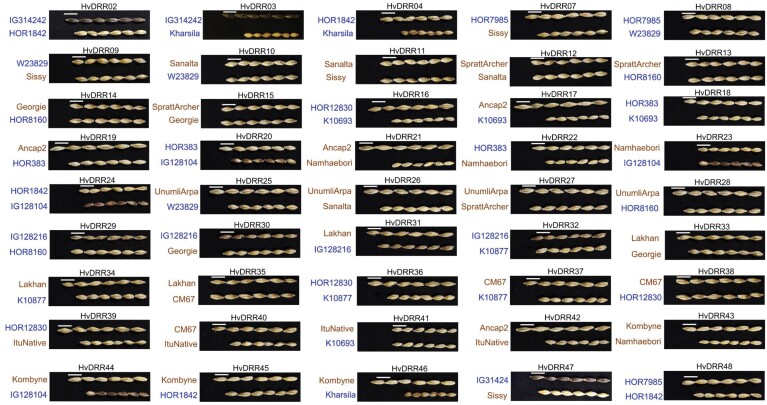
Images of grains of parental inbreds of 45 HvDRR sub-populations. The white scale bar indicates a length of 10 mm. Brown and blue coloured text designates germplasm type cultivars and landraces, respectively.

We used 45 RIL populations developed from the genetic crosses among 23 spring barley inbreds (Casale *et al.*, 2022). The inbred lines used to develop the HvDRR population were selected from the barley diversity panel described by (Haseneyer *et al.*, 2010) to maximize a genotypic and trait diversity index (Weisweiler *et al.*, 2019). The 23 parental inbreds were crossed in a double round-robin design. Each inbred was crossed with four other inbreds (Fig.1; Supplementary Fig. S1). From each cross, a RIL population was developed following the single seed descent principle.

### Field experiment and data collection

Each RIL was grown in field experiments from 2017 to 2019, and seed size characteristics data were collected from Quedlinburg in 2018, and Cologne from 2017 to 2019, amounting to four different environments. We followed an augmented incomplete block design with one replicate for each RIL in the four environments. The RILs from a single HvDRR sub-population were sown in neighbouring rows with 30 seeds per genotype in a 160 cm row. The parental inbreds were replicated as repeated checks across 16 to 20 rows randomized across the entire trial at each environment. Border plots were also maintained surrounding the entire trial. The ears were harvested and dried for a minimum of 3 d before threshing. MARViN seed analyser (MARViNTECH GmbH, Germany) was used to estimate the seed size parameters for a random subset of around 60 seeds from each row of the field experiment. The camera mounted on the device recorded the image of the seed spread on the imaging platform. The seed image was then analysed by the in-built software that returned seed size characteristics, including the count of seeds on the platform, GL in mm, GW in mm, and GA in mm^2^. Furthermore, TGW was measured for all genotypes.

### Genotyping

In the F_4_ generation, 35–146 RILs from each of the 45 HvDRR sub-populations (Supplementary Fig. S1) were genotyped using a 50 K barley single-nucleotide polymorphism (SNP) array (Bayer *et al.*, 2017). The genetic map for individual HvDRR sub-populations, and consensus map across the HvDRR population were established as described by Casale *et al.* (2022), and used for genomic prediction and linkage mapping.

### Statistical analyses

Data were processed and statistical analysis was performed using R platform (R core team 2021). A correction of field heterogeneity was performed at each environment based on the field map of the augmented design using the R software’s mvngGrAd package (Technow 2016). Adjusted entry means for original and field heterogeneity, corrected values were estimated by using a mixed linear model with fixed genotype effects, and random location and genotype: location effects:


$$Y_{ijk}=\;\mu +G_{i}+E_{i}+{\left(G:E\right)}_{ij}+\;\varepsilon_{ijk},$$

where $Y_{ijk}$ was the observed phenotypic value for $i^{th}$ genotype in $j^{th}$ environment for $k^{th}$ replication, before or after field correction for the augmented design,

μ the general mean,

$G_{i}$ the effect of $i^{th}$ genotype,

$E_{i}$ the effect of $j^{th}$ environment,

${\left(G:E\right)}_{ij}$ the interaction of $i^{th}$ genotype with $j^{th}$ location and

$\varepsilon_{ijk}$ the random error term.

For broad sense heritability ($H^{2}$ ) estimation on entry mean basis, the variance component of $\sigma_{g}^{2}$ was calculated based on the above model, but with a random genotype effect, and the following method was used:


$$eqnarrayH^{2}=\sigma_{g}^{2}/(\sigma_{g}^{2}+\frac{c}{2}),$$

where *c* is the mean variance of a difference of two adjusted entry means (Piepho and Möhring, 2007).

The $H^{2}$ across environments was highest for non-field heterogeneity adjusted data (Supplementary Table S1). Therefore, the adjusted entry means across the four environments were calculated from the original data without field heterogeneity correction. Variance components were tested using a restricted likelihood ratio test using the R package RLRsim (Scheipl 2022).

In order to examine the mean differences between the segregating populations and the respective parental inbreds, the following mixed model was fitted to the data of all progenies and the two parental inbreds from all four environments:


$$y_{ijk}=\mu +GT_{l}+G_{il}+E_{j}+{\left(G:E\right)}_{ij}+\;\varepsilon_{ijk},$$

where $GT_{l}$ is the fixed effect of the germplasm type (either parental inbred or progeny).

### Genomic prediction

Genome-wide predictions of all phenotypes were performed according to Inghelandt *et al.* (2019) by genomic best linear unbiased prediction (GBLUP) using the following model (VanRaden, 2008):


y=1μ+Zu+ε,

where *y* was the vector of the adjusted entry means of the corresponding phenotype, 1 the unit vector, *μ* the general mean, *Z* the design matrix that assigned the random effects to the genotypes and, *u* the vector of the genotypic effects that were assumed to be normally distributed with $u\sim N\left(0,K\sigma_{u}^{2}\right)\;$ , where *K* denotes the realized kinship matrix considering only additive effects calculated based on the SNP marker data mentioned above and $\sigma_{u}^{2}\;$ being the genotypic variance of the GBLUP model. In addition, ε was the vector of residuals following a normal distribution $\varepsilon \sim N\left(0,I\sigma_{\varepsilon }{}^{2}\right)$ , where *I* was the identity matrix and $\sigma_{\varepsilon }{}^{2}$ the residual variance.

Prediction ability was calculated as the Pearson correlation coefficient between observed and predicted phenotypes $r\left(y,\hat{y}\right)$ . Five–fold cross-validations (CVs) with 20 replications were performed to assess the model performance. The prediction ability was defined as the median of the prediction abilities across the 20 runs of 5-fold CV.

### QTL analysis

In the first step, QTLs were detected in the entire HvDRR population using the multi-parent population (MPP) procedure of the mppR package (Garin *et al.*, 2017). The MPP QTL analysis used a consensus map of the 45 HvDRR sub-populations. Next, the parental model was applied to estimate the multi-QTL effect, with the assumption that each parent contributes a unique allele and the effect of a parent is constant across all crosses involving the parent in question. In short, the QTL detection procedure involved a simple interval mapping (SIM) in identifying the associated markers. Then, markers detected from the SIM scan were used as cofactors to run a first composite interval mapping. Finally, a second composite interval mapping scan was run using a backward elimination procedure to obtain a final list of QTLs for the grain size characteristics.

In the second step, we also performed a single population QTL analysis (SPA) for grain size and TGW in 45 individual HvDRR sub-populations using the R package qtl (Broman and Sen, 2009). First, genotype probabilities of every 1 cM were estimated, followed by a genome-wide scan for marker-trait association using Haley-Knott regression approximation. A forward search algorithm was then applied to perform multiple QTL mapping. A permutation test of 4000 runs was performed, and the logarithm of the odds (LOD) score at a 0.05 significance level was set as a threshold for QTL detection. A 1.5 LOD drop sets the confidence interval on either side of the detected QTL (Dupuis and Siegmund, 1999). The SPA QTLs detected across the HvDRR sub-populations were summarized as consensus QTLs. For the consensus QTLs, the shortest possible confidence interval of co-located QTLs (across sub-populations and phenotypes) was used to define the interval of consensus QTLs. The confidence interval remained the same in the case of QTLs detected in only one sub-population for a single seed size or weight character.

Finally, a two-dimensional genome-wide scan was performed to detect epistatic loci. The LOD score for interaction at a 0.05 significance level obtained from the permutation test of 1000 runs was set as a threshold for epistatic QTL detection.

### Allelic series estimate

To understand the contribution of the parental inbred to the genetic makeup of grain size and weight, we considered the effect sizes of the above-described MPP QTL analysis. First, a multiple comparison test was performed on each parental inbred allelic effect across all MPP QTLs. Next, we calculated the cumulative allele effect for each inbred as the sum of the negative or positive standardized allele effects. The standardized allele effect for an inbred was the difference between the mean of the estimated allele effect for the 23 parental inbreds and the estimated allele effect of the corresponding inbred.

### QTL validation

We developed a high-resolution mapping population that segregated for a GL-associated QTL on chromosome 1H by crossing two RILs from the HvDRR33 sub-population, namely Hv-2018S-7-03050 and Hv-2018S-7-03225, harbouring Lakhan and Georgie allele, respectively. Georgie and Lakhan are the parental inbreds of the HvDRR33 sub-population. Cleaved amplified polymorphic sequence markers were developed for genotyping the parent-specific SNP allele at the left border (JHI-Hv50k-2016-43007) and right border (JHI-Hv50k-2016-43714) of the QTL interval for 924 F_2_ progenies (Supplementary Table S2). DNA was extracted from 1-week-old seedlings for genotyping, and the SNP loci at left and right borders were PCR amplified. Then, the PCR product on the left and right border were digested using *Xba*I and *Bsp*EI, respectively. Digested products were separated on a 1.5 % agarose gel and visualized under a UV-transilluminator. Recombinants were selected and transferred to 1.5 l pots. The grains were harvested, and GL was evaluated using MARViN seed analyser. We also assessed GL from three non-recombinant F_2_ plants, each carrying either Lakhan or Georgie alleles at both borders of the QTL interval.

### Candidate gene analysis

A detailed candidate gene analysis was performed for the consensus QTLs smaller than 10 Mb, that explained more than 10% of the phenotypic variance. The list of high confidence genes in the consensus QTLs were retrieved from the plant genomics and phenomics research data repository, IPK Gatersleben, Germany (https://edal-pgp.ipk-gatersleben.de/). The annotation and coordinates were based on the Morex v3 gene models (Mascher *et al.*, 2021). In the next step, we used the variant calling data obtained from the whole genome sequencing project of the 23 inbreds to select the genes that showed polymorphisms between the parental inbreds (Weisweiler *et al.*, 2022). We examined non-synonymous (amino acid substitutions) or deleterious synonymous mutations, indels in the coding region, as well as indels and structural variants (SVs) in the 5-end regulatory region (5 kb upstream of the start codon). The genes with polymorphisms between the parental groups with distinct allele effects on grain size and TGW at consensus QTLs were considered candidate genes. Finally, Sanger sequencing was used to validate the mutations detected in the variant calling data for two candidate genes.

## Results

### Phenotypic variation for grain size and weight

**Fig. 2.**
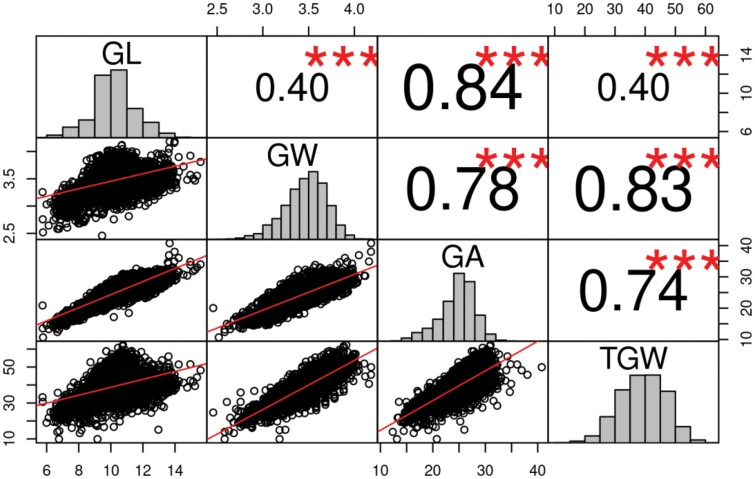
Pairwise correlation coefficients between four grain size characteristics, grain area (GA), grain length (GL), grain width (GW), and thousand-grain weight (TGW). Adjusted entry means of the recombinant inbred lines from 45 individual HvDRR sub-populations and the parental inbreds were used for estimating the correlation. Asterisks indicate that the observed correlation coefficient estimates were statistically different from zero using the Pearson correlation coefficient test (***P≤0.001). The size of the text corresponds to the magnitude of correlation coefficient estimates.

Grain size and weight phenotypes were collected across four different environments for ~4000 RILs from the 45 sub-populations. The parental inbreds were replicated between 16–20 times across the experiment in each environment as repeated checks to account for field heterogeneity. We observed significant (*P*<0.0001) genotype, environment, and genotype×environment effects for all four characteristics GW, GL, GA and TGW (Table 1). On an entry mean basis, broad-sense heritability was 84 % or above for all characteristics (Table 1). Although a significant positive correlation was observed between all grain size characteristics, the degree of association between TGW and GW (r=0.84) was 2–fold higher than with GL (r=0.4; Fig. 2). In addition, we observed contrasting relations among the grain size characteristics for some sub-populations compared with the whole HvDRR population. For instance, GL showed no or weak correlation with GW and TGW for HvDRR18 and HvDRR32. In general, GA showed significant positive correlations with all characteristics at the whole HvDRR population as well as sub-populations level (Supplementary Fig. S2; Fig. 2).

**Table 1.** Variance components and broad-sense heritability (*H*^*2*^) of grain size-related characteristics and thousand-grain weight (TGW).

Characteristic	Genotype	Environment	Genotype:Environment	Residual	*H* ^*2*^
Grain length	1.53***	0.57***	0.24***	0.56***	0.84
Grain width	0.04***	0.02***	0.01***	0.01***	0.85
Grain area	9.95***	0.16***	0.92***	2.43***	0.89
TGW	46.34***	20.92***	7.42***	17.72***	0.84

Asterisks indicate the significance of a likelihood ratio test (***P≤0.001).

The adjusted entry means for GL and GW ranged from 5.7–15.6 mm and 2.5–4.2 mm, respectively, among the RILs of HvDRR sub-population (Supplementary Figs S3, S4). Likewise, the lowest and highest observed adjusted entry means for GA were 10.7 mm^2^ and 40.2 mm^2^, respectively (Supplementary Fig. S5). The adjusted entry means for TGW ranged between 10 and 62.3 g (Supplementary Fig. S6). We also compared, across all environments, the average progeny performance with the parental average for 45 HvDRR sub-populations, as this indicates the importance of additive×additive epistatic variance. Mean progeny characteristic values for GL, GW, GA, and TGW were statistically different (*P*<0.01) from the parental average in 16, 16, 18, and 17 HvDRR sub-populations for GL, GW, GA, and TGW, respectively. Among them, seven sub-populations showed significantly (*P*<0.01) different average parental and progeny means for all four characteristics (Supplementary Figs S3-S6). Overall, we observed a considerable genotypic variation for grain size and weight in the HvDRR population that can be used for breeding and genetic dissection of grain size, and therewith yield-related characteristics in barley.

### QTLs associated with grain size

**Fig. 3.**
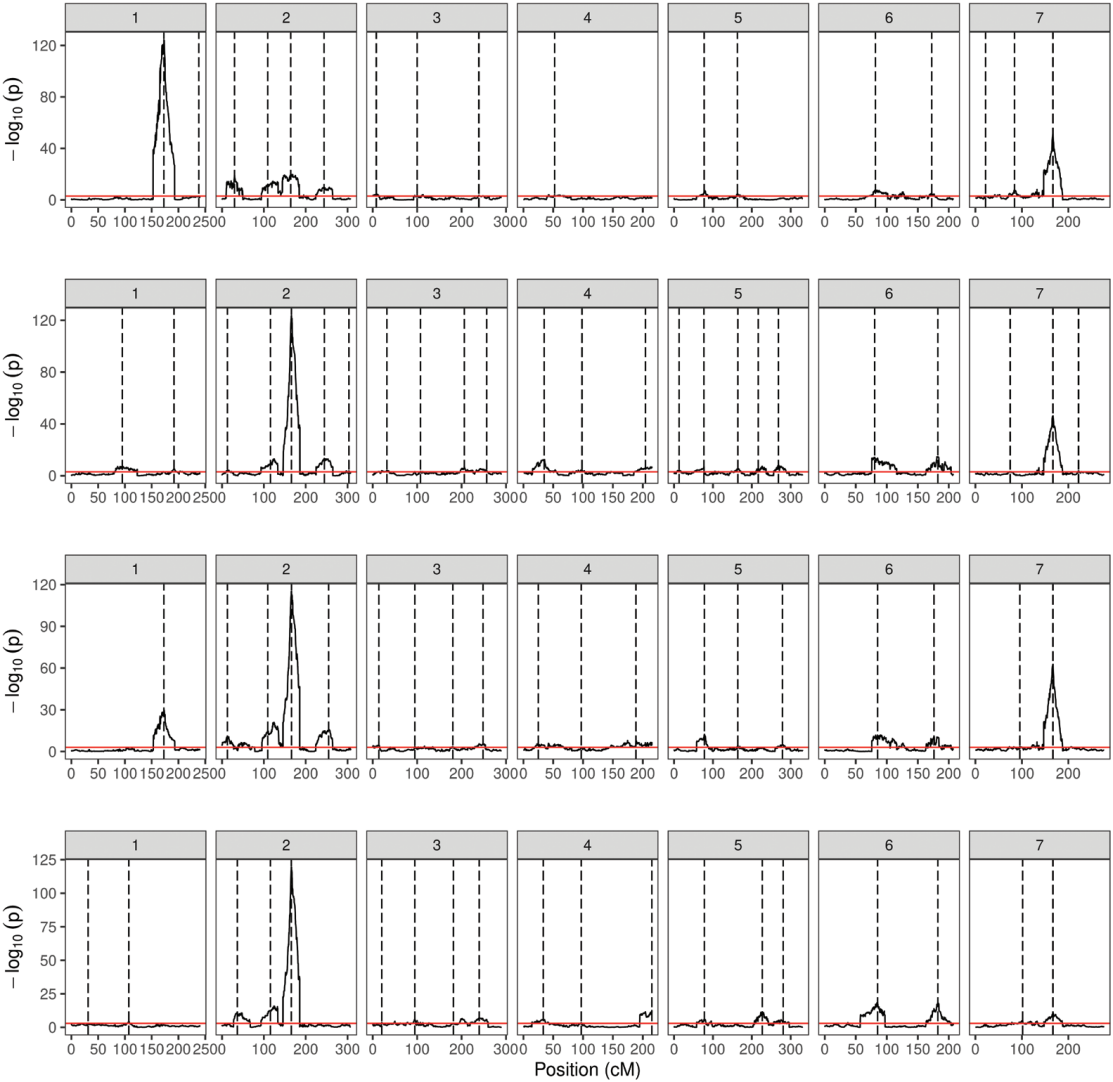
Quantitative trait loci (QTLs) profile for grain size-related characteristics from multi-parent population analysis of 45 HvDRR sub-populations, using the parental model. QTL profile for (A) grain length, (B) grain width (C), grain area, and (D) thousand-grain weight. The QTLs were detected across all seven chromosomes using composite interval mapping [significant threshold of –log10(p)=3]. The vertical dashed lines indicate the positions of the detected QTL.

First, we performed MPP analyses that detected 17, 24, 19, and 19 QTLs associated with GL, GW, GA, and TGW, respectively. Nine of these QTLs were associated with all four evaluated characteristics (Fig. 3). The full model of additive QTLs in MPP explained 41.6%, 46.9%, 45.7% and 40.4% of the phenotypic variance for GL, GW, GA, and TGW, respectively (Table 2). Likewise, the percentage of phenotypic variance (r² of prediction ability) explained by the GBLUP model based on genome-wide SNPs was 69.6 % (GW), 71.7 % (GL) and 73.4 % (GA and TGW; Table 3).

**Table 2.** Percent of explained phenotypic variance by the full model of additive quantitative trait loci (QTLs) detected in multi-parent population analysis using a parental model.

Characteristic	Explained phenotypic variance (%)
Grain length	41.6
Grain width	46.9
Grain area	45.7
Thousand-grain weight	40.4

**Table 3.** Prediction ability of the genome-wide SNP marker data for the grain size-related characteristics and thousand-grain weight (TGW).

Characteristic	Prediction ability overall 45 HvDRR populations		Standard deviation
	Without CV	5-Fold CV 20 runs	5-Fold CV 20 runs
Grain length	0.919	0.847	0.012
Grain width	0.919	0.834	0.012
Grain area	0.932	0.856	0.012
TGW	0.92	0.857	0.009

The predictions were performed using genomic best linear unbiased predictions. The second column indicates the prediction abilities without cross-validation (CV). The third and fourth columns are the medians of prediction ability, and SD across 20 runs of 5-fold CV made with random sampling, respectively.

**Fig. 4.**
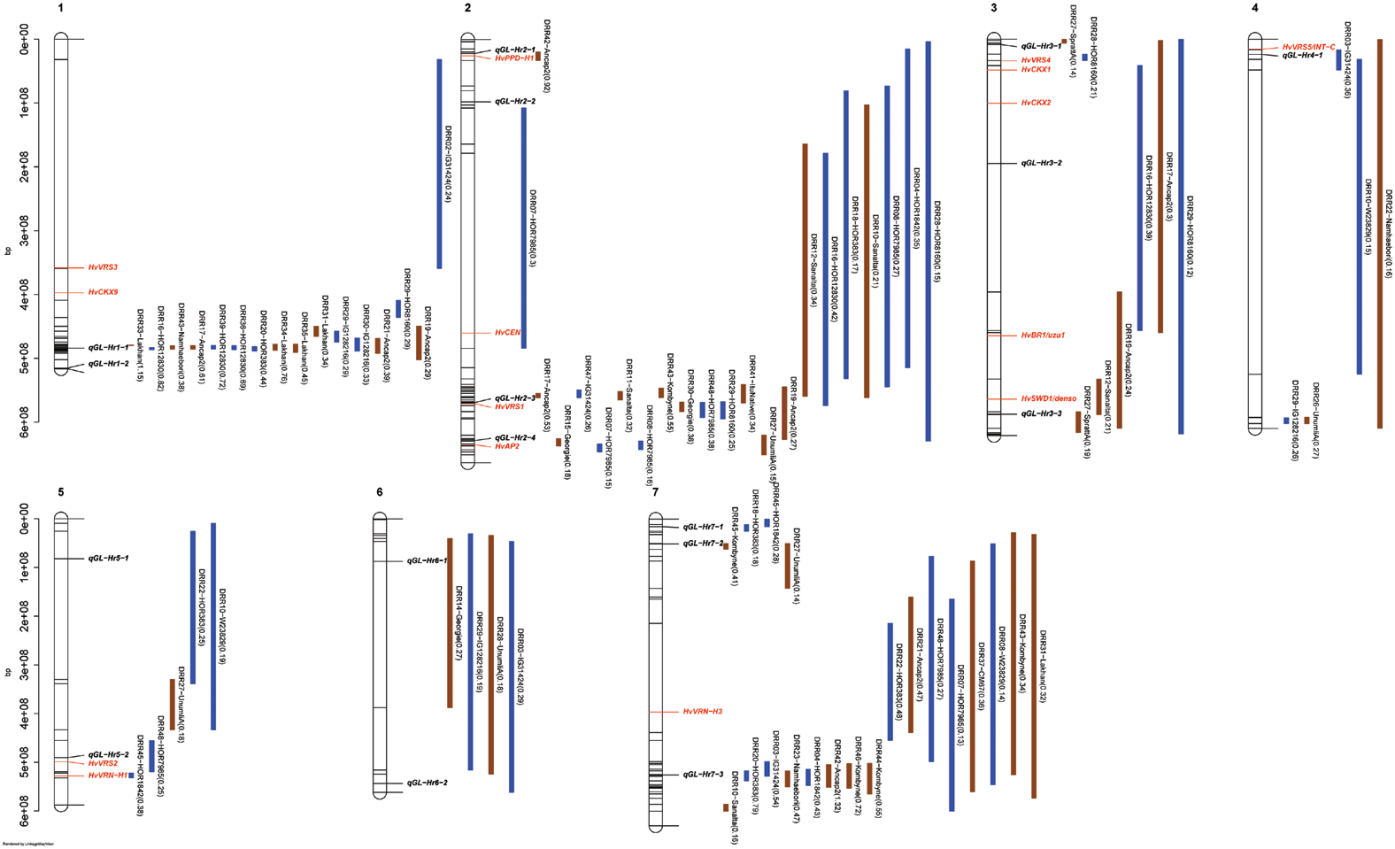
Confidence intervals of quantitative trait loci (QTLs) detected for grain length (GL) in single population analyses. QTL mapping was performed in 45 HvDRR sub-populations. The parental allele with a positive additive effect inside the parenthesis is connoted in the QTL name. The colour of the confidence interval implies if the parent contributing a positive additive effect is a landrace (blue) or a cultivar (brown). The position of known genes controlling developmental phenotypes such as flowering time, plant height, and spikelet branching are indicated in red letters. The loci in bold italics letters are the GL-associated QTLs detected in multi-parent population analysis using the parental model. A consensus map was used to order the marker for plotting the confidence intervals. The physical positions of the top and bottom five markers of the genetic map, and the markers flanking the QTL interval are indicated in the plot.

**Fig. 5.**
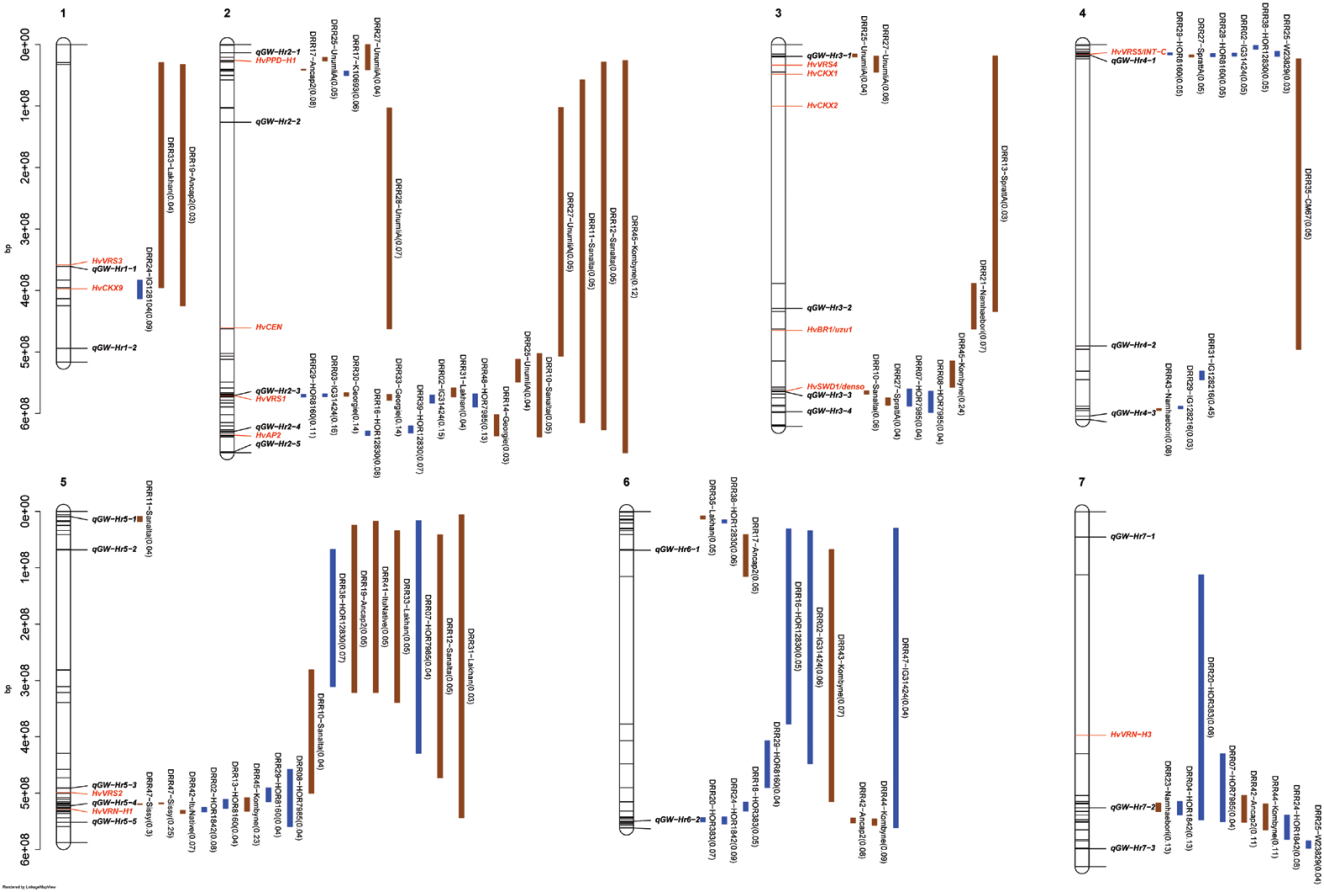
Confidence intervals of quantitative trait loci (QTLs) detected for grain width (GW) in single population analyses. QTL mapping was performed in 45 HvDRR sub-populations. The parental allele with a positive additive effect inside the parenthesis is connoted in the QTL name. The colour of the confidence interval implies if the parent contributing a positive additive effect is a landrace (blue) or a cultivar (brown). The position of known genes controlling developmental phenotypes such as flowering time, plant height, and spikelet branching are indicated in red letters. The loci in bold italics letters are the GW-associated QTLs detected in multi-parent population analysis using the parental model. A consensus map was used to order the marker for plotting the confidence intervals. The physical positions of the top and bottom five markers of the genetic map, and the markers flanking the QTL interval are indicated in the plot.

**Fig. 6.**
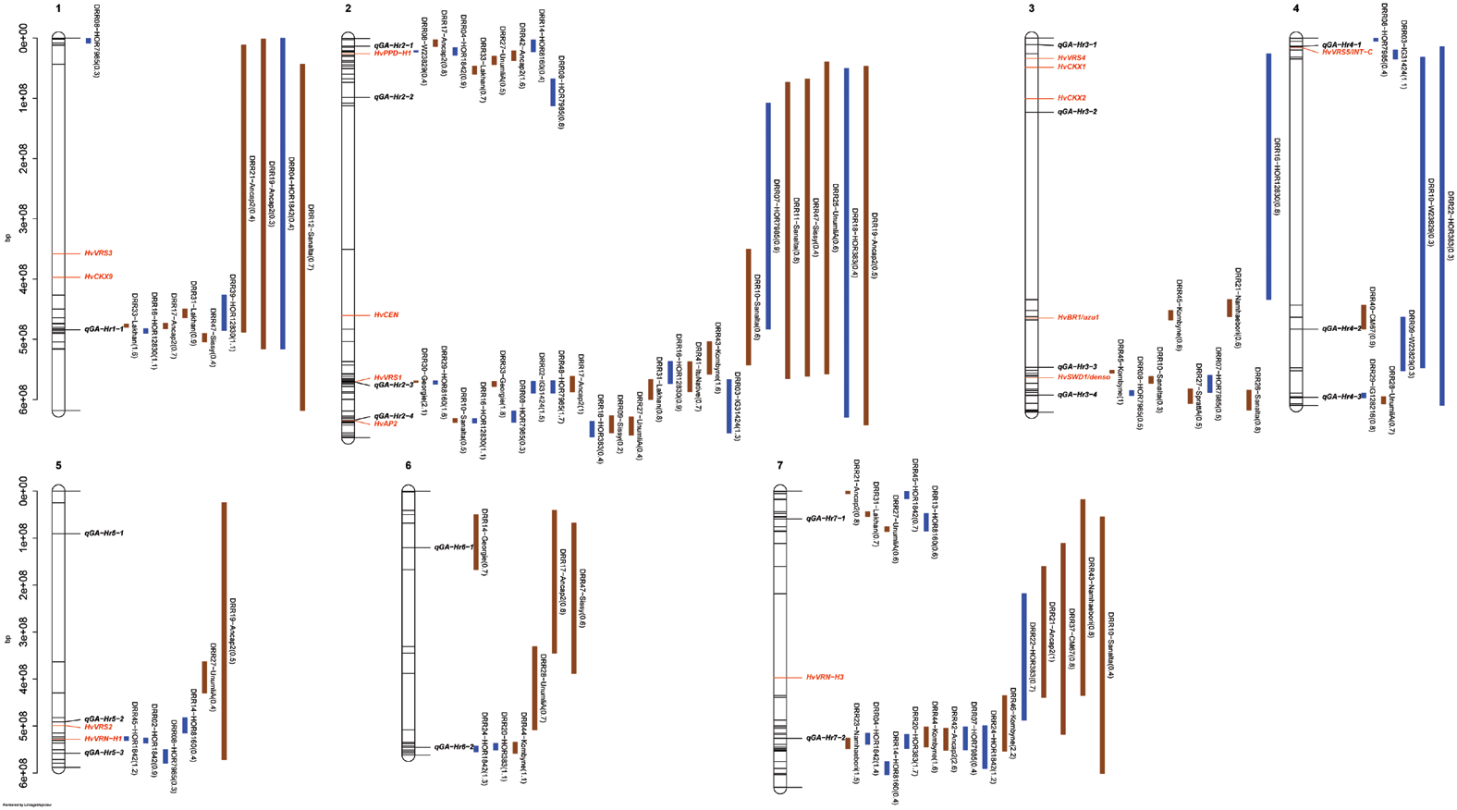
Confidence intervals of quantitative trait loci (QTLs) detected for grain area (GA) in single population analyses. QTL mapping was performed in 45 HvDRR sub-populations. The parental allele with a positive additive effect inside the parenthesis is connoted in the QTL name. The colour of the confidence interval implies if the parent contributing a positive additive effect is a landrace (blue) or a cultivar (brown). The position of known genes controlling developmental phenotypes such as flowering time, plant height, and spikelet branching are indicated in red letters. The loci in bold italics letters are the GA-associated QTLs detected in multi-parent population analysis using the parental model. A consensus map was used to order the marker for plotting the confidence intervals. The physical positions of the top and bottom five markers of the genetic map, and the markers flanking the QTL interval are indicated in the plot.

**Fig. 7.**
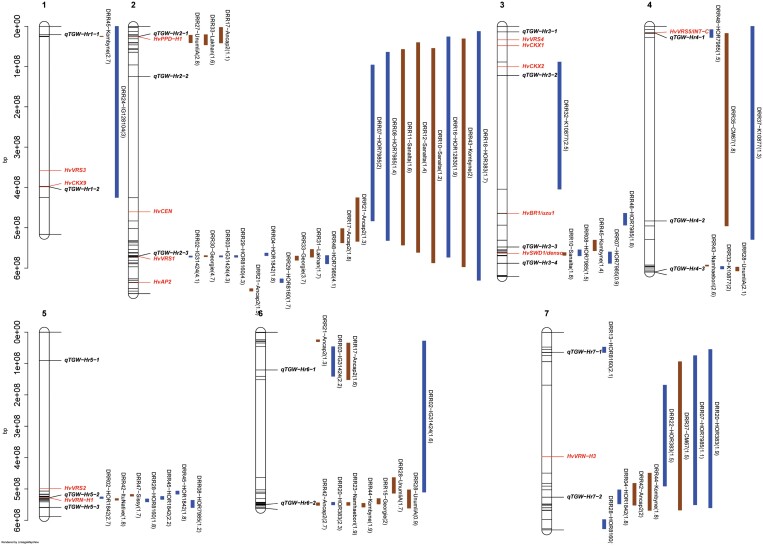
Confidence intervals of quantitative trait loci (QTLs) detected for thousand-grain weight (TGW) in single population analyses. QTL mapping was performed in 45 HvDRR sub-populations. The parental allele with a positive additive effect inside the parenthesis is connoted in the QTL name. The colour of the confidence interval implies if the parent contributing a positive additive effect is a landrace (blue) or a cultivar (brown). The position of known genes controlling developmental phenotypes such as flowering time, plant height, and spikelet branching are indicated in red letters. The loci in bold italics letters are the TGW associated QTLs detected in multi-parent population analysis using the parental model. A consensus map was used to order the marker for plotting the confidence intervals. The physical positions of the top and bottom five markers of the genetic map, and the markers flanking the QTL interval are indicated in the plot.

In SPA, we detected QTLs in 38, 35, 39, and 33 of the HvDRR sub-populations for GL, GW, GA, and TGW, respectively (Supplementary Tables S3-S6). A total of 316 QTLs for four characteristics were detected in SPA, of which 260 were observed at the same position as MPP QTLs (Figs 4-7). When examining the detailed distribution of SPA QTLs, 99 and 56 were located on chromosomes 2H and 7H, respectively, and the number of QTLs ranged between 29 and 36 for the other chromosomes (Supplementary Table S3-S6). The percentage of explained variance (PEV) by individual QTLs in SPA ranged between 3–70% (Supplementary Tables S3-S6). More than two-thirds of the QTLs detected in our study explained 10% or more of the phenotypic variance for the associated characteristic (Supplementary Fig. S7). Finally, we summarized the SPA QTLs into 62 consensus QTLs, the shortest possible interval of co-located QTLs in SPA (Supplementary Table S7). Only six consensus QTLs were present in the pericentromeric region.

As explained above, we observed a significant difference between progeny and parental mean for a few sub-populations and, thus, evaluated the presence of epistatic QTLs (Supplementary Table S8). We detected 14 significant (*P*<0.05) epistatic interaction QTLs. The epistatic interaction QTLs were frequently detected (10 times) in sub-populations with significant differences (*P*<0.01) for the grain size and weight characteristics between parent and population mean, compared with sub-populations without that feature (Supplementary Table S8).

### QTL effect estimate

**Fig. 8.**
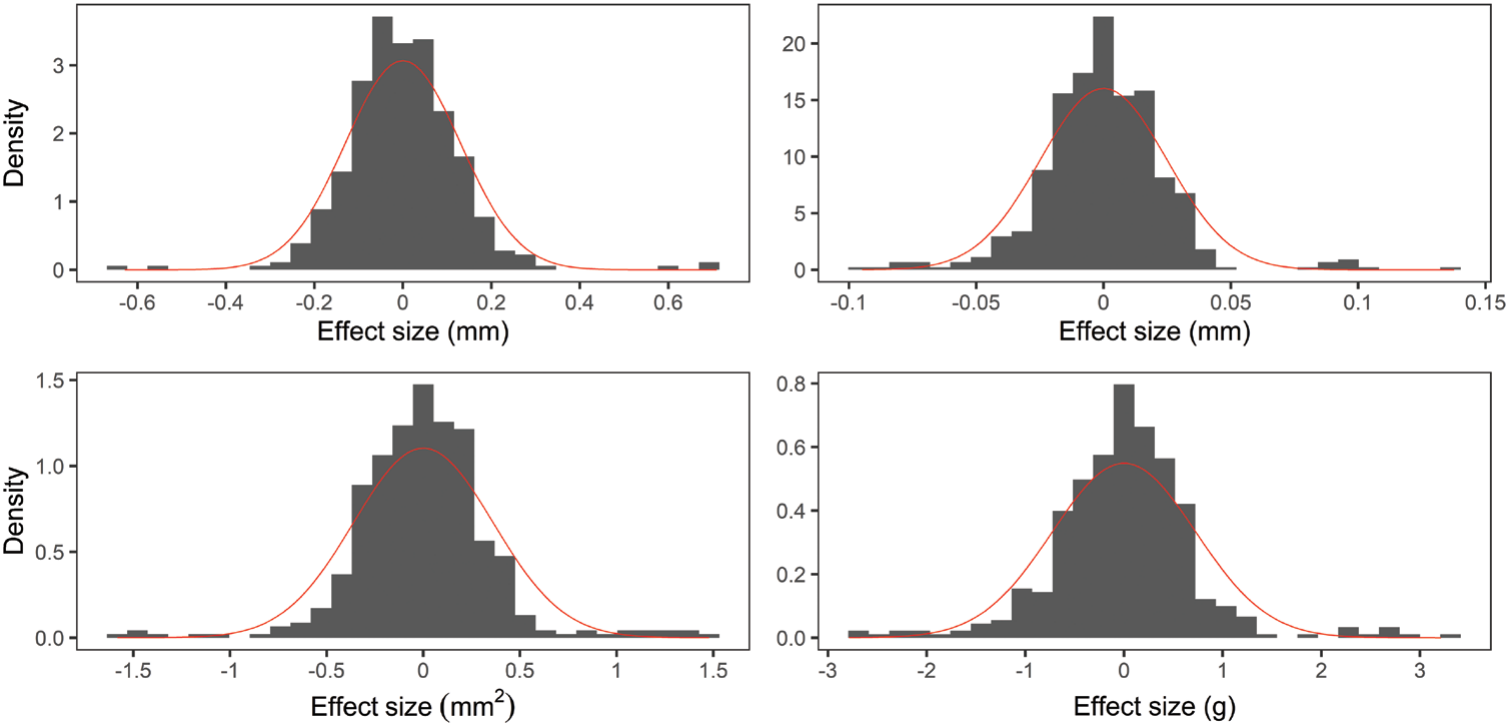
Histogram of standardized allele effect estimates for quantitative trait loci (QTLs) detected in multi-parent population analysis of 45 HvDRR sub-populations using a parental model for (A) grain length, (B) grain width, (C) grain area, and (D) thousand-grain weight. The standardized allele effect for an inbred was the difference between the mean of the estimated allele effects for the 23 parental inbreds and the estimated allele effect of the considered inbred. A normal distribution curve (red colour) is overlaid onto the histograms.

To understand the contribution of parental inbreds to the genetic makeup of sink size and grain weight, we estimated the effect size contributed by parental inbreds across all QTLs detected in MPP QTL analysis using a parental model. That means one MPP QTL for a characteristic will have 23 alleles corresponding to 23 parental inbreds, resulting in total QTL counts of 391, 552, 437, and 437 for GL, GW, GA, and TGW, respectively (Fig. 8). The distribution of the QTL allele effect indicated that the differences in grain size and weight of 23 parental inbreds were caused by the cumulative effect of minor-effect alleles and a few large-effect alleles (Fig. 8). Only 10% of the QTL alleles caused TGW to increase or decrease by 1 g or more (Fig. 8D). Similarly, 4% of the QTLs affected GL, GW, and GA by more than 2.5 mm, 0.5 mm and 1 mm^2^, respectively (Fig. 8A-C).

**Fig. 9.**
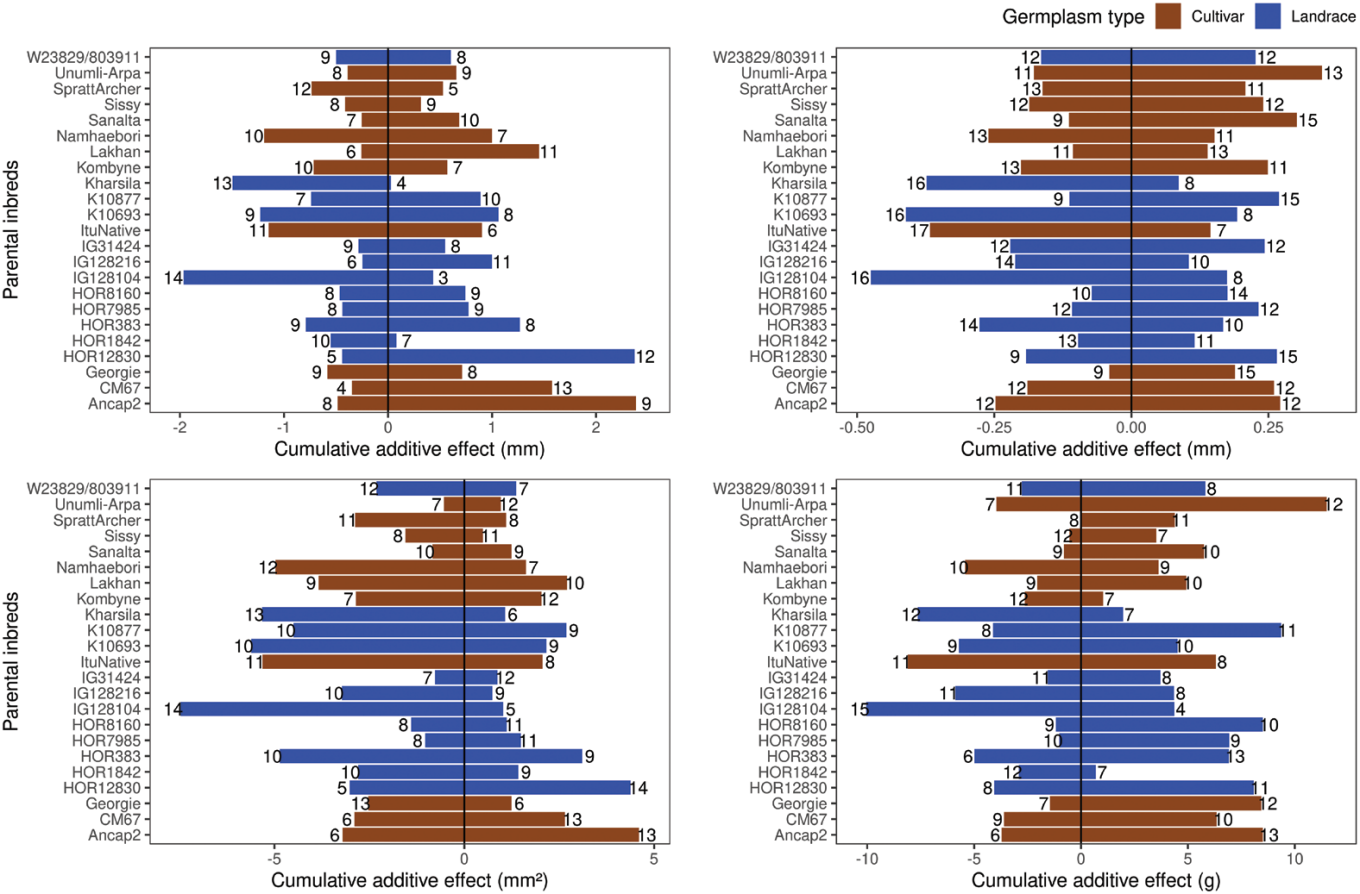
Cumulative allele effect estimate for quantitative trait loci (QTLs) detected in multi-parent population analysis of 45 HvDRR sub-populations using a parental model. Sum of positive (right box) and negative (left box) standardized allele effect size for 23 inbreds stacked across (A) grain length, (B) grain width, (C) grain area, and (D) thousand-grain weight QTLs. The cumulative allele effect was the sum of the standardized allele effect for an inbred, where the standardized allele effect for an inbred was the difference between the mean of the estimated allele effects for 23 parental inbreds and the estimated allele effect of the considered inbred. The number next to the bars indicate the count of QTLs with negative or positive standardized allele effect.

Next, allelic series across the MPP QTLs revealed that parental inbreds contributed to both positive and negative allele effects (increase or decrease in grain size; Supplementary Figs S8-S11). In addition, the allele effect of parental inbreds was statistically different within a single QTL (Supplementary Figs S8-S11). Because we observed the allelic series for parental inbreds, we estimated the count and the cumulative positive and negative allele effect across the MPP QTLs for each parental inbred (Fig. 9). The net cumulative allele effect (cumulative decrease subtracted from cumulative increase) and the number of favourable alleles were lowest for a landrace IG128104 across the MPP QTLs for all four characteristics, followed by another landrace Kharsila. On the other hand, across GL and GA QTLs, the HOR12830 (1.76 mm and 2.73 mm^2^) and Ancap2 (1.68 mm and 2.88 mm^2^) allele contributed to a higher net cumulative effect than the other parental inbreds. Sanalta, Unumli-Arpa, and K10877 were the most significant contributors to the number of favourable alleles, and the cumulative positive allele effect across GW QTLs detected in MPP analysis (Fig. 9).

The average allele count of positive and negative effects across MPP QTLs was almost the same for landraces and cultivars across all four characteristics. However, landraces had higher cumulative negative and lower cumulative positive effects than cultivars (Fig. 9). For example, the average allele count for negative allele effect for TGW was 9.1 and 10.2 for landraces and cultivars, respectively. Nevertheless, the average cumulative negative effect for landraces (–4.3 g) was 1.5 fold greater than for cultivars (–2.9 g). It was noteworthy that alleles from landraces (HOR12830, K10877, HOR8160, and HOR7985) also contributed to a high genome-wide count of positive allele effects as well as cumulative allele effects (Fig. 9).

### Candidate genes associated with grain size

We obtained information for SNP annotations, indels, and predicted SVs from the whole-genome sequencing of the 23 parental inbreds. This information was used to compare the sequence polymorphism between the parental inbreds of HvDRR populations in the coding and regulatory region of the genes present in the confidence intervals of 18 consensus QTLs with an interval length <10 Mbp. The genes in the consensus QTLs that showed sequence polymorphisms in the coding region and the 5’-end regulatory region between the parental inbreds with distinct allele effects on grain size characteristics were selected as candidate genes. As a result, we identified 965 candidate genes for these 18 consensus QTLs. The number of candidate genes for the consensus QTLs varied between 7 and 151 (Supplementary Table S9).

Among 62 consensus QTLs, 14 loci harboured the closest barley orthologs of genes controlling grain size in other crops (Supplementary Tables S7, S9, S10). Among them, we identified allelic variations in the regulatory or coding region for eight orthologs. Furthermore, these polymorphisms corresponded to the parental allele effect on QTLs detected across the sub-populations (File 1 at Zenodo https://zenodo.org/record/6497793; Shrestha *et al*., 2022).

**Fig. 10.**
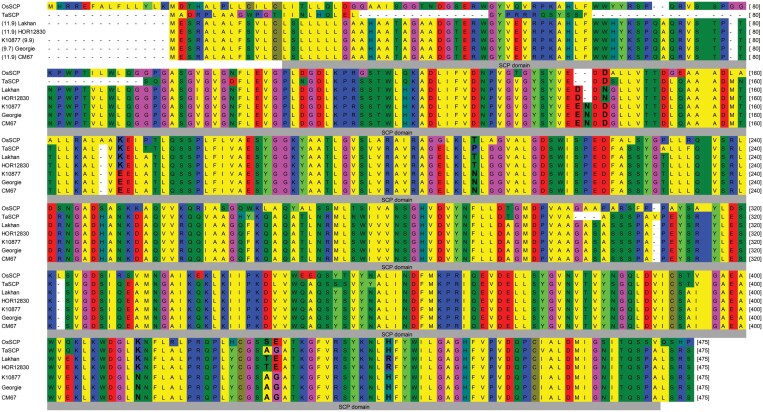
Candidate gene underlying *qHvDRR-GS-6* on chromosome 1H. Protein alignment of a candidate gene *HORVU.MOREX.r3.1HG0078020* encoding carboxypeptidase domain protein among the five parental inbreds of the HvDRR population with orthologs of rice (*OsSCP*) and wheat (*TaSCP*). SNPs and a 3 bp indel caused amino acid substitutions in the conserved serine carboxypeptidase domain (indicated with grey box). The protein sequence of the barley gene was highly conserved in rice and wheat. Lakhan and HOR12830 alleles at the QTL contributed to longer grains. Sites in bold letters indicate amino acid substitutions between parental groups, with contrasting allele effects at the *qHvDRR-GS-6*.

*qHvDRR-GS-6* was one of the major effect QTLs for GL with PEV from 25-62% in SPA. Further, we validated *qHvDRR-GS-6* in a fine-mapping population derived from the cross of two RILs from the HvDRR33 sub-population carrying Lakhan (long grains) and Georgie (short grains) alleles at this QTL. We found 35 recombinants (bearing different parental alleles at the left and right border of the QTL interval) among 924 F_2_ progenies. The phenotyping of GL in the F_2_ population distinguished the two classes of non-recombinants, with Lakhan and Georgie allele-bearing progenies producing long and short grains, respectively. The average GL for different groups of recombinants ranged from 8.6–9.2 mm (Supplementary Fig. S12). Although only the initial steps of the positional cloning of the underlying gene for *qHvDRR-GS-6* were done in the current study, the consensus interval harboured genes annotated as carboxypeptidase (*HORVU.MOREX.r3.1HG0078020*) and TCP transcription factor (*HORVU.MOREX.r3.1HG0077830*). The variant calling data indicated amino acid substitution polymorphisms between Lakhan and Georgie in the two candidate genes mentioned above. The protein sequence of both candidate genes was highly conserved among other monocots (Fig. 10; Supplementary Fig. S13).

## Discussion

In the present study, we demonstrated the application of the HvDRR population of barley to identify the genetic factors controlling grain size characteristics. We observed that the broad-sense heritability (*H*^*2*^) on an entry mean basis was more than 84% for all characteristics (Table 1), indicating a substantial genetic variability for grain size in the HvDRR population. With 95%, a slightly higher *H*^*2*^ was reported by Wang *et al.* (2019) for grain size characteristics in a double haploid population of barley. The *H*^*2*^ for GW, GA, and TGW observed for the HvDRR population was comparable to that observed for the HEB-25 NAM population (Maurer *et al.*, 2015) grown under adequate nitrogen fertilization (Sharma *et al.*, 2018). At the same time, *H*^*2*^ for GL was more than 2-fold higher in our study than in the HEB-25 NAM population (Sharma *et al.*, 2018). The latter observation might be because the HEB-25 NAM population was derived from a cross between Barke and wild barley genotypes. In such populations, GL might be overestimated by awn retention due to threshability problems (Schmalenbach *et al.*, 2011), leading to lower heritability.

Although the maximum (4.2 mm) and minimum (2.5 mm) GW observed in RILs of the HvDRR population was similar to previous reports, we observed a considerably broader range of adjusted entry means for GL, GA, and TGW. For instance, the minimum and maximum GL on the adjusted entry mean basis among all RILs was 5.7 and 15.6 mm, respectively. On the other hand, the range for mean GL reported earlier for barley segregating populations was 7.4–11 mm (Walker *et al.*, 2013; Zhou *et al.*, 2016; Sharma *et al.*, 2018; Wang *et al.*, 2019; Watt *et al*., 2019, 2020). The above observation can be explained by the fact that most studies (except Sharma *et al.*, 2018) comprised a single bi-parental population and the genetic variance is restricted to two parents. In addition, the parental inbreds of the HvDRR population were selected to maximize genotypic and phenotypic diversity (Weisweiler *et al.*, 2019). Therefore, TGW in the HvDRR population showed about the same range as worldwide spring barley (Pasam *et al.*, 2012). These observations support the results of earlier simulation studies (Stich, 2009; Klasen *et al.*, 2012), that partial diallel designs as implemented in our study capture more allelic diversity than NAM type populations, thus, providing high power for unravelling the genetics of natural phenotypic variation.

### Genetic complexity of grain size variation

We performed MPP and SPA analyses and detected 79 and 316 grain size and weight QTLs, respectively (Figs 3-7). In general, the LOD scores of QTLs detected in MPP analysis were higher than SPA, which suggested an increased QTL detection power in MPP analysis (Garin *et al.*, 2021). However, 56 SPA QTLs were not detected at the same positions as MPP QTLs (Figs 4-7) because the rare allele with a small effect detected in specific sub-populations might not have passed the significance threshold in MPP analysis. In addition, MPP analyses might be affected by potential imprecisions in the consensus map; hence, we considered the results of both MPP and SPA in our study.

In order to do so, we summarized the SPA QTLs into 62 consensus QTLs, the shortest possible confidence interval of co-located QTLs across sub-populations or characteristics (Supplementary Table S7). The number of consensus QTLs associated with grain size and weight in the HvDRR population was considerably higher than in previous reports (Supplementary Table S7). For instance, in our study, 42 and 36 consensus QTLs were linked to GA and TGW, respectively (Supplementary Table S7). In contrast, only seven and 11 GA QTLs were detected in the studies of Wang *et al.* (2019) and Sharma *et al.* (2018), respectively. Xu *et al.* (2018) identified 21 GA-associated QTLs, which was half of the number detected in the HvDRR population. The same trend was observed for GL, GW, and TGW. When comparing the physical interval of the consensus QTLs with the QTLs detected in previous linkage and association mapping studies concerning grain size and weight, 13 consensus QTLs for grain size characteristics detected in our study have not been previously reported. These findings suggest that grain characteristics in barley are inherited in a more complex manner than previously reported.

In addition, we observed that the prediction ability of the genomic selection model was about 0.85 (Table 3), which was 20% higher than the comparable figure for a QTL-based prediction, i.e. the square root of the PEV of the full additive model of MPP QTLs (Table 2). This finding suggests that even with a big mapping population like ours, the effect of many more minor effect loci did not pass the significant threshold in MPP analyses. Thus, the actual genetic complexity of grain size characteristics is even more complex than discussed above.

Despite the high genetic complexity of the studied characteristics across all HvDRR populations, we have observed that PEV by the majority (~0.6 frequency) of the SPA QTLs was 10–20 % also after cross-validation (File 2 at Zenodo). This finding indicates that QTL fine mapping and, ultimately, map-based cloning of the underlying genes, will be possible for a considerable proportion of the detected QTLs when the most relevant sub-populations are studied, and identification of such sub-populations was one of the objectives of this study.

### Previously described QTLs for grain parameters detected in the HvDRR population

We detected major effect genes associated with spikelet architecture and hulled caryopsis in the HvDRR population as a proof of concept (Supplementary Tables S7, S9; Supplementary Fig. S13). For example, the consensus QTL *qHvDRR-GS-18* was detected in six two-rowed versus six-rowed sub-populations and harboured *Vrs1* locus (Supplementary Table S7). Sequence comparisons of the parental inbred lines revealed that six-rowed and two-rowed barley carried *vrs1* (mutant allele) and *Vrs1* (wild type allele), respectively (Supplementary Fig. S14). *Vrs1* (homeobox domain-containing protein) is the major gene determining the lateral spikelet fertility in barley and has a pleiotropic effect on grain size (Komatsuda *et al.*, 2007). Therefore, the bigger and heavier grain in two-rowed genotypes may compensate for fewer seeds compared with six-rowed genotypes (Ayoub *et al.*, 2002).

Likewise, *qHvDRR-GS-61* was detected at the *Nud* locus in HvDRR populations involving naked and hulled barley (Supplementary Table S7). This observation indicated a pleiotropic effect of naked caryopsis on grain size and weight, which was also reported by Wang *et al.* (2019) in a mapping population developed from naked and hulled barley. Parental inbreds of the HvDRR population with the non-adhering hull (Kharsila, IG128104) revealed a 17 kb deletion (7H:525 620 758-525 637 446), resulting in the null allele for an ethylene response factor gene which is characteristic of naked barley genotypes (Taketa *et al.*, 2008).

Furthermore, we confirmed the QTL allele effect in a high-resolution population segregating for one of the novel GL QTLs (*qHvDRR-GS-6*) located on chromosome 1H (Supplementary Fig. S12). These examples illustrated the accuracy and relevance of the detected loci in our study, and suggested that the detailed consideration of the other detected QTLs provides biologically relevant information.

### The function of some grain size-related genes might be conserved in cereals

We used the SNP, indels, and predicted SVs of the 23 parental inbreds in coding and potentially regulatory regions (Weisweiler *et al.*, 2022) and their functional annotation to describe the underlying candidate genes (Supplementary Table S9). We detected polymorphisms in the coding region among the contrasting parental inbreds for the genes present in the consensus QTLs. For instance, the barley ortholog of *GS5* (*HvGS5*) was the candidate for *qHvDRR-GS-24* associated with all four evaluated grain characters (File 1 at Zenodo). *GS5* encodes a serine carboxypeptidase protein and is described as a positive regulator of grain size and weight in rice and wheat (Li *et al.*, 2011; Ma *et al.*, 2016; Wang *et al.*, 2016). Another gene (*HORVU.MOREX.r3.1HG0078020*) closely related to *HvGS5* was the most plausible candidate for *qHvDRR-GS6* (Supplementary Table S9).

In addition, *qHvDRR-GS6* harboured a barley gene (*HORVU.MOREX.r3.1HG0077830*) which is closely related to *TCP5-*like transcription factor family of proteins. The latter regulates leaf and petal size in Arabidopsis (van Es *et al.*, 2018). We detected amino acid substitutions or indels in *HORVU.MOREX.r3.1HG0078020* and *HORVU.MOREX.r3.1HG0077830* between parental alleles with long (Lakhan and HOR12830) and short grains (Georgie, K10877 and CM67) at *qHvDRR-GS6* (Fig. 1; Supplementary Table S9). We further validated the non-synonymous mutations and exon indels identified from the whole-genome sequencing using Sanger sequencing of both genes. These results indicated the accuracy of the variant calling file used to select the candidate gene in our study (Fig. 10; Supplementary Fig. S13). The above genes should be targeted for functional validation studies.

The barley ortholog for *TaCYP78A3* (Ma *et al.*, 2015) encoding a cytochrome P450 (*HvCYP78A*) was the candidate gene for *qHvDRR-GS-58*. Similarly, a gene closely related to *HvCYP78A HORVU.MOREX.r3.2HG0106460* on chromosome 2H was the most promising candidate for *qHvDRR-GS-11* (Supplementary Tables S7, S9 and File 1 at Zenodo). Cytochrome P450 is a large protein family involved in different biosynthetic processes (Li *et al.*, 2019). For example, CYP78A, one of the cytochrome P450 family members, is a positive regulator of seed size in wheat and Arabidopsis (Nagasawa *et al.*, 2013; Ma *et al.*, 2015). Likewise, *HvGSK2* (*q-HvDRR-GS-3*), *HvWG7* (*q-HvDRR-GS-14*), *HvSRS5* (*q-HvDRR-GS-33*), *HvLGY3* (*q-HvDRR-GS-36*) and *HvRGA1* (*q-HvDRR-GS-55*) were the other barley orthologs of grain size associated genes from rice detected for the respective QTLs (Supplementary Tables S7, S9 and File 1 at Zenodo).

*HORVU.MOREX.r3.5HG0507280*, a gene encoding E3 ubiquitin-protein ligase, might be the causal gene for *qHvDRR-GS-43*. It revealed amino acid substitutions among the parental lines (Supplementary Table S9). *GW2* in rice encodes an E3 ubiquitin-protein ligase involved in the ubiquitin-proteasome signalling pathway (Song *et al.*, 2007). It is a negative regulator of grain size and the function is conserved in monocots, including rice, wheat, and maize (Song *et al.*, 2007; Li *et al.*, 2010a; Simmonds *et al.*, 2016). Genes encoding strictosidine synthase was the candidate for *qHvDRR-GS-45* (*HORVU.MOREX.r3.5HG0510870*). Strictosidine synthase showed indole-3-acetic acid glucose hydrolase activity and negatively regulated TGW in rice (Ishimaru *et al.*, 2013). We also detected genes encoding the bi-directional sugar transporter SWEET (*HORVU.MOREX.r3.6HG0628390*) as the candidate gene for *qHv-DRR-54* on chromosome 6H (Supplementary Table S9). The SWEET family protein is involved in seed filling in Arabidopsis. Triple knockout mutant of a SWEET family protein (*sweet11;12;15*) in Arabidopsis produced wrinkled seeds with reduced weight (Zhou *et al.*, 2015). Different classes of zinc finger proteins, receptor kinases, and transcription factors (MADS-box, MYB) were the other categories of highly represented candidate genes that are involved in seed development (Supplementary Table S9). In conclusion, candidate gene analysis identified genes that are known to be involved in cell proliferation, cell elongation and grain filling, which ultimately determines grain size and weight.

### Sink size primarily determines grain weight in the HvDRR population

We measured GL, GW, and GA as a proxy of sink size in our study. Across the HvDRR population, the correlation between these characters and TGW was up to r=0.84 (Fig. 2). This suggests that variability in grain filling can only explain a low additional proportion of the variability of grain weight, and that sink size is the primary determinant of grain weight in the HvDRR population. In addition, of the 62 consensus QTLs detected in our study, 14 and 11 loci were associated exclusively with GL and GW, respectively but not with TGW (Supplementary Table S7). Likewise, 35 consensus QTLs were linked to TGW, and only three were not associated with any of the grain size parameters (Supplementary Table S7). This supported our above conclusion that sink size was the major determinant of grain weight in the HvDRR population. Our observation agrees with the findings of Bingham *et al.* (2007), that the mean grain weight of field-grown barley was strongly determined by potential grain size.

Similar to previous studies (Sharma *et al.*, 2018; Xu *et al.*, 2018; Wang *et al.*, 2019), we observed a stronger correlation between TGW and GW than between TGW and GL in the HvDRR population (Fig. 2). This might be explained by the order in which GW and GL develop. It has been shown in wheat that GL reaches its maximum at the initial grain filling period, whereas GW reaches a maximum at the late grain filling period in wheat. Another explanation might be that the increase in grain volume occurs in the transverse plane during the rapid grain filling stage, which will more directly affect GW than GL (Xie *et al.*, 2015). Due to the genetic relatedness between barley and wheat, the change in grain dimension post-anthesis might be similar, explaining a better correlation between TGW and GW compared with TGW and GL, which warrants further investigation.

Across the entire HvDRR population, a significant positive correlation between GL and GW was observed (Fig. 2). However, in agreement with earlier studies (Sharma *et al.*, 2018; Watt *et al*., 2019, 2020), in seven sub-populations (HvDRR13, HvDRR14, HvDRR18, HvDRR19, HvDRR22, HvDRR26, and HvDRR32), we observed a particularly weak correlation between GL and GW (Supplementary Fig. S2). This observation suggested that GL and GW are at least partially genetically independent. The QTL detected in SPA of the above-mentioned sub-populations for GL did not overlap with those for GW (Figs 4, 5; Supplementary Tables S3, S4), suggesting that different genes contribute to their variation. GL-associated QTLs without effect on GW and vice versa were also observed in other cereals such as rice (Yin *et al.*, 2020).

In addition, a total of 15 consensus QTLs were detected for both GL and GW (Supplementary Table S7). Among them, the allele effect was in the same direction for GL and GW for 13 QTLs (Supplementary Tables S3, S4). This trend was also reported for most of the grain size QTLs in rice (Song *et al.*, 2007; Che *et al.*, 2016; Xiao *et al.*, 2019; Zhong *et al.*, 2020) and wheat (Bednarek *et al.*, 2012; Ma *et al.*, 2015; Liu *et al.*, 2020). For two of the QTLs, namely *qHvDRR-GS-45* and *qHvDRR-GS-52*, the QTL effects revealed an opposite direction for GL and GW (Supplementary Tables S3, S4, S7). *qHvDRR-GS-45* and *qHvDRR-GS-52* did not harbour the orthologs of rice genes for *GS9* (Zhao *et al.*, 2018) and *GW5* (Li *et al.*, 2011) that regulate the development of short and wide grains. Therefore, identifying the underlying genes in barley is particularly of interest to maximize yield.

### Beneficial alleles for grain size in barley landraces

The favourable allele for increased grain size and uniform shape is accumulated in elite germplasm because of directional selection during the domestication process (Gegas *et al.*, 2010; Zhang *et al.*, 2021). However, landraces that evolved from natural and human selection might still be a significant source of positive genetic diversity (Casañas *et al.*, 2017). To this date, linkage mapping studies for grain size in barley were limited to genetic crosses between cultivars or cultivars and wild relatives (Zhou *et al.*, 2016; Sharma *et al.*, 2018; Wang *et al*., 2019, 2021; Watt *et al*., 2019, 2020). However, the parental inbreds of the HvDRR population comprised 12 landraces and 11 cultivars. We compared the allelic count and the cumulative allele effect across QTLs detected in MPP analysis between landraces and cultivars. The net allelic count with a positive effect size for grain size and weight QTLs detected from MPP analysis was slightly higher for cultivars (54%) than for landraces (46%). In addition, the net cumulative allele effect (difference between cumulative positive and negative effects) was slightly higher for cultivars than for landraces (Fig. 9). The finding that a higher proportion of alleles from cultivars contributes to an increase in grain size and weight indicates that bigger and heavier grains were positively selected in barley in recent breeding history. This is in agreement with findings that in rice for most cloned grain size QTLs such as *GS3, GW5, GW2, GW8, GW7, TGW3,* and *TGW6,* the wild-type alleles were the negative regulators of grain size (Li *et al.*, 2019).

However, the result of the allelic series analysis across the MPP QTLs implied that all parental inbreds contributed positive and negative allele effects (Supplementary Figs S8-S11). This finding was in accordance with the results of Sharma *et al.* (2018), who reported that the effect of 25 wild barley alleles segregating in cultivated barley showed both positive and negative allele effects across the QTL hotspots for different grain size characteristics. Nevertheless, within a single QTL, the effect of all wild barley alleles primarily showed the same direction. In contrast, we identified statistically significant (*P*<0.05) groups of parental inbreds with a differing magnitude of allele effect across the respective QTL (Supplementary Figs S8-S11), regardless of being cultivars or landraces. It is particularly advantageous because HvDRR sub-populations involving parental inbreds on the extreme of the allelic series at a QTL might be ideal for fine-mapping of the locus. The allelic series data also indicated that landraces such as K10877, HOR12830, HOR8160, and HOR7985 made significant contributions to favourable alleles across the grain size and weight QTLs (Fig. 9). For instance, the allelic series comparison at MPP QTLs revealed that landraces contributed to higher positive effects at *qGL-Hr1-1, qGL-Hr7-2, qGW-Hr5-4, qGW-Hr7-3, qTGW-Hr5-1,* and *qTGW-Hr5-2*, compared with cultivars (Supplementary Figs S8, S9, S11). This observation is in accordance with the finding that some domestication alleles of grain size genes in rice (Liu *et al.*, 2017a), maize (Li *et al.*, 2010b), and wheat (Liu *et al.*, 2020) were also detected in the primary gene pool.

The results of this study indicate that the genetic architecture of grain size is more complex than previously reported. Although the grain size and weight phenotypic variation were mainly controlled by the additive effect of minor and moderate effect alleles, we also detected a few major effect alleles for grain size and weight characteristics. Allelic series across the MPP QTLs indicated that all 23 parental inbreds have positive and negative allele effects on grain size and weight. The net cumulative allele effect was only slightly higher for cultivars than landraces, illustrating the potential of the latter for breeding projects. We used the whole genome sequencing data of 23 parental inbreds and identified several promising candidate genes. Future work will focus on validating these candidate genes and the performance of yield trials using RILs that carry the alleles with a positive effect. This study demonstrates the utility of the HvDRR barley population for dissecting the genetic architecture of complex characteristics, and welcomes the scientific community to exploit the resources.

## Supplementary data

The following supplementary data are available at *JXB* online.

Table S1. Broad-sense heritability of grain size-related traits.

Table S2. Primers used in the study.

Table S3. Summary of quantitative trait loci (QTLs) detected in HvDRR sub-populations for grain length.

Table S4. Summary of quantitative trait loci (QTLs) detected in HvDRR sub-populations for grain width.

Table S5. Summary of quantitative trait loci (QTLs) detected in HvDRR sub-populations for grain area.

Table S6. Summary of quantitative trait loci (QTLs) detected in HvDRR sub-populations for thousand-grain weight.

Table S7. Consensus quantitative trait loci (QTLs) associated with grain size and weight in the HvDRR population.

Table S8. Summary of genome-wide epistatic loci detected in HvDRR population.

Table S9. Candidate genes present in the consensus intervals.

Table S10. The barley orthologs of genes associated with seed size in other plant species.

Fig. S1. The double round-robin (DRR) crossing scheme used to establish the HvDRR population.

Fig. S2. Pairwise correlation analysis between the four evaluated traits.

Fig. S3. Boxplot of adjusted entry means of the recombinant inbred lines of the HvDRR populations for grain length.

Fig. S4. Boxplot of adjusted entry means of the recombinant inbred lines of the HvDRR populations for grain width.

Fig. S5. Boxplot of adjusted entry means of the recombinant inbred lines of the HvDRR populations for grain area.

Fig. S6. Boxplot of adjusted entry means of the recombinant inbred lines of the HvDRR populations for thousand-grain weight.

Fig. S7. Distribution of the percentage of variance explained by quantitative trait loci detected in single population analyses.

Fig. S8. Multiple comparisons of the standardized allele effect for grain length.

Fig. S9. Multiple comparisons of the standardized allele effect for grain width.

Fig. S10. Multiple comparisons of the standardized allele effect for grain area.

Fig. S11. Multiple comparisons of the standardized allele effect for thousand-grain weight.

Fig. S12. QTL allele effect validation at *qHvDRR-GS-6* on chromosome 1H.

Fig. S13. Protein alignment of a candidate gene underlying *qHvDRR-GS-6* on chromosome 1H.

Fig. S14. The allelic variant for *vrs1* (*vulgare six-rowed spike 1*).

erac369_suppl_Supplementary_Figures_S1-S14

erac369_suppl_Supplementary_Tables_S1-S9

## Data Availability

The adjusted entry means of the recombinant inbred lines from 45 HvDRR sub-populations are deposited at Zenodo (https://zenodo.org/record/6497793; Shrestha *et al*., 2022). The information to retrieve genetic maps and variant calling data can be obtained from Casale *et al.* (2022) and Weisweiler *et al.* (2022). The codes implemented for data processing (field heterogeneity correction and general linear model), single population QTL analysis, multi-parent population QTL analysis and epistatic QTL models are deposited at https://github.com/s7ashre/qtlmapping. The codes implemented to prepare graphs can be provided upon request. Requests for seeds of RILs from the HvDRR population or sub-populations can be sent to the corresponding author.

## Abbreviations

DRR: double-round robin
GA: grain area
GBLUP: genomic best linear unbiased prediction
GL: grain length
GW: grain width
GWAS: genome-wide association study
LOD: logarithm of the odds
MPP: multi-parent population
PEV: percent of explained variance
RIL: recombinant inbred line
SNP: single-nucleotide polymorphism
SPA: single population analysis
SV: structural variant
QTL: quantitative trait locus
TGW: thousand-grain weight
