# Supplementary material for: The double round-robin population unravels the genetic architecture of grain size in barley
Source: J Exp Bot. 2022 Sep 12;73(22):7344–61. doi: 10.1093/jxb/erac369 (PMC9730814; doi:10.1093/jxb/erac369)
Supplement: erac369_suppl_Supplementary_Figures_S1-S14 [file erac369_suppl_supplementary_figures_s1-s14.pdf]

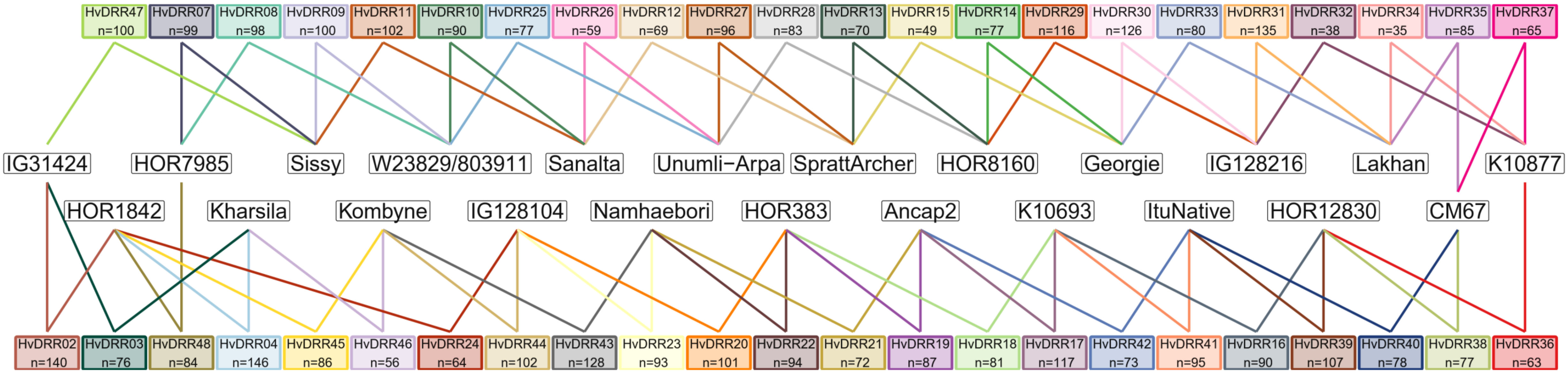

Fig. S1. The double round-robin (DRR) crossing scheme used to establish the HvDRR population. Parental inbred lines for each individual HvDRR sub-population are connected by the lines and the ‘n’ indicates the number of recombinant inbred lines (Casale *et al.,* 2022).

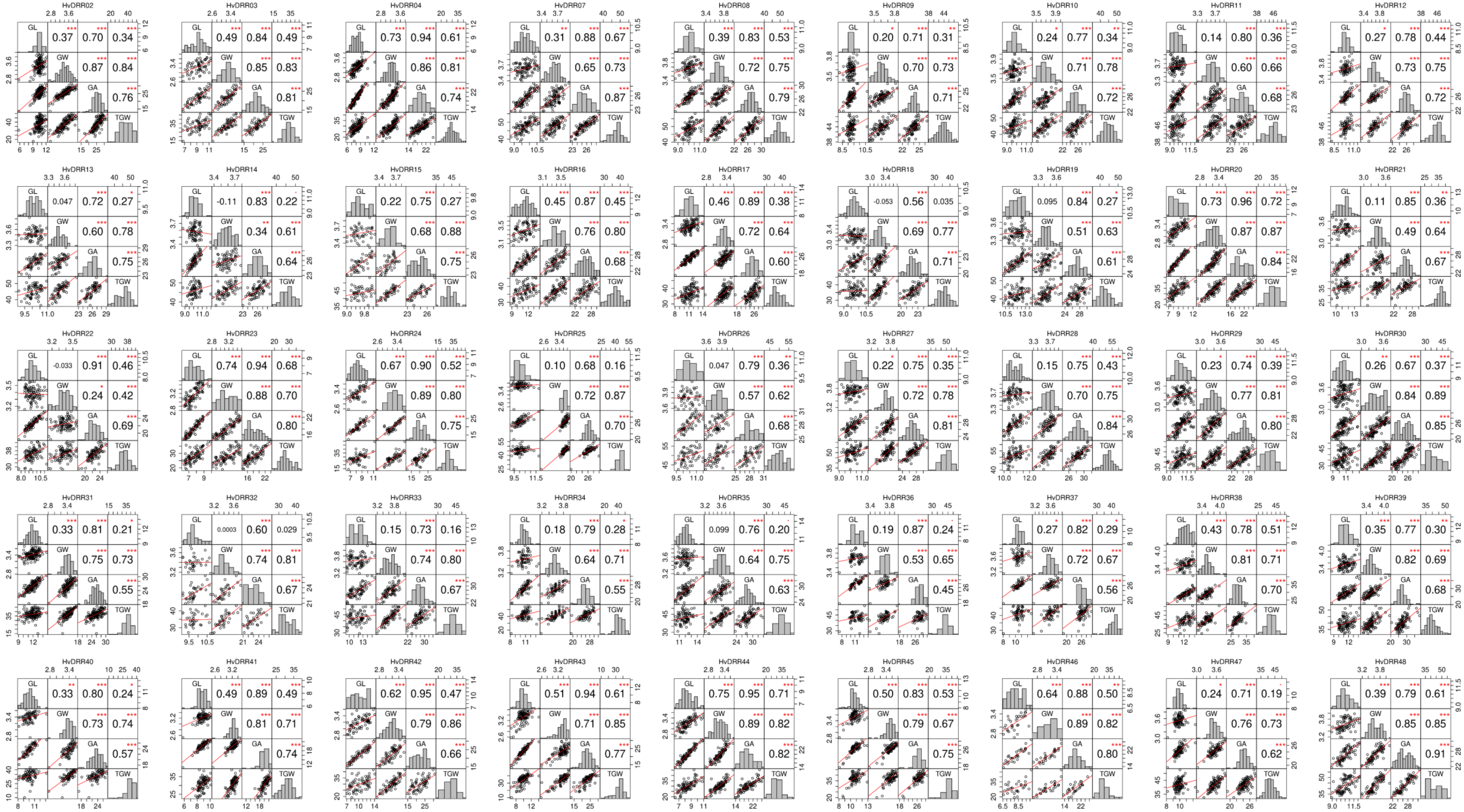

Fig. S2. Pair-wise correlation analysis between the four evaluated traits namely grain length (GL) in mm, grain width (GW) in mm, grain area (GA) in mm^2 and thousand grain weight (TGW) in g across 45 HvDRR sub-populations.

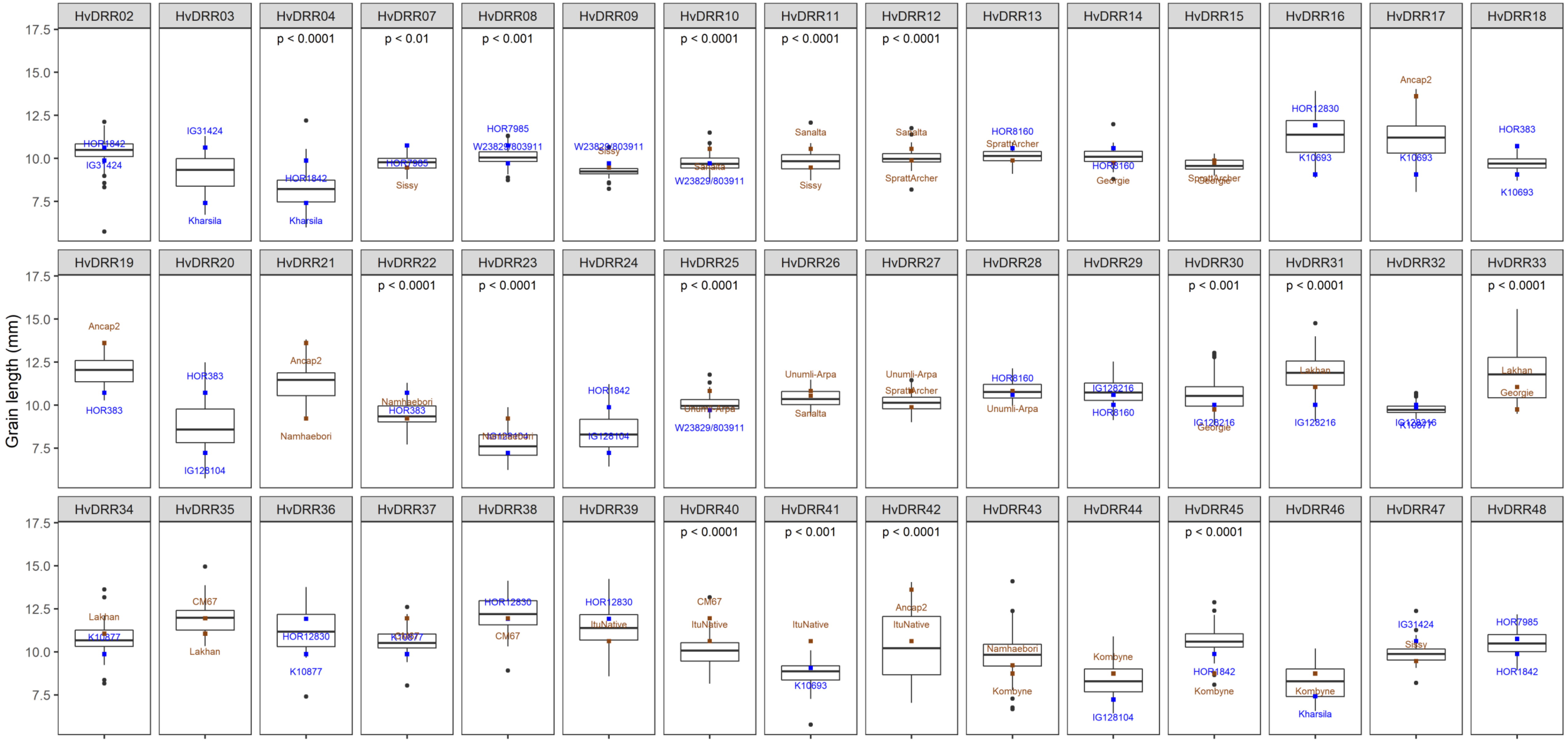

Fig. S3. Boxplot of adjusted entry means of the recombinant inbred lines of the HvDRR populations for grain length in mm. The square dots indicate the adjusted entry mean of the parental inbreds of the respective recombinant inbred line populations. Brown and blue colors designate germplasm-type cultivars and landraces, respectively. The p-value above the boxplot indicates a significant mean differences between the segregating populations and the respective parental inbreds.

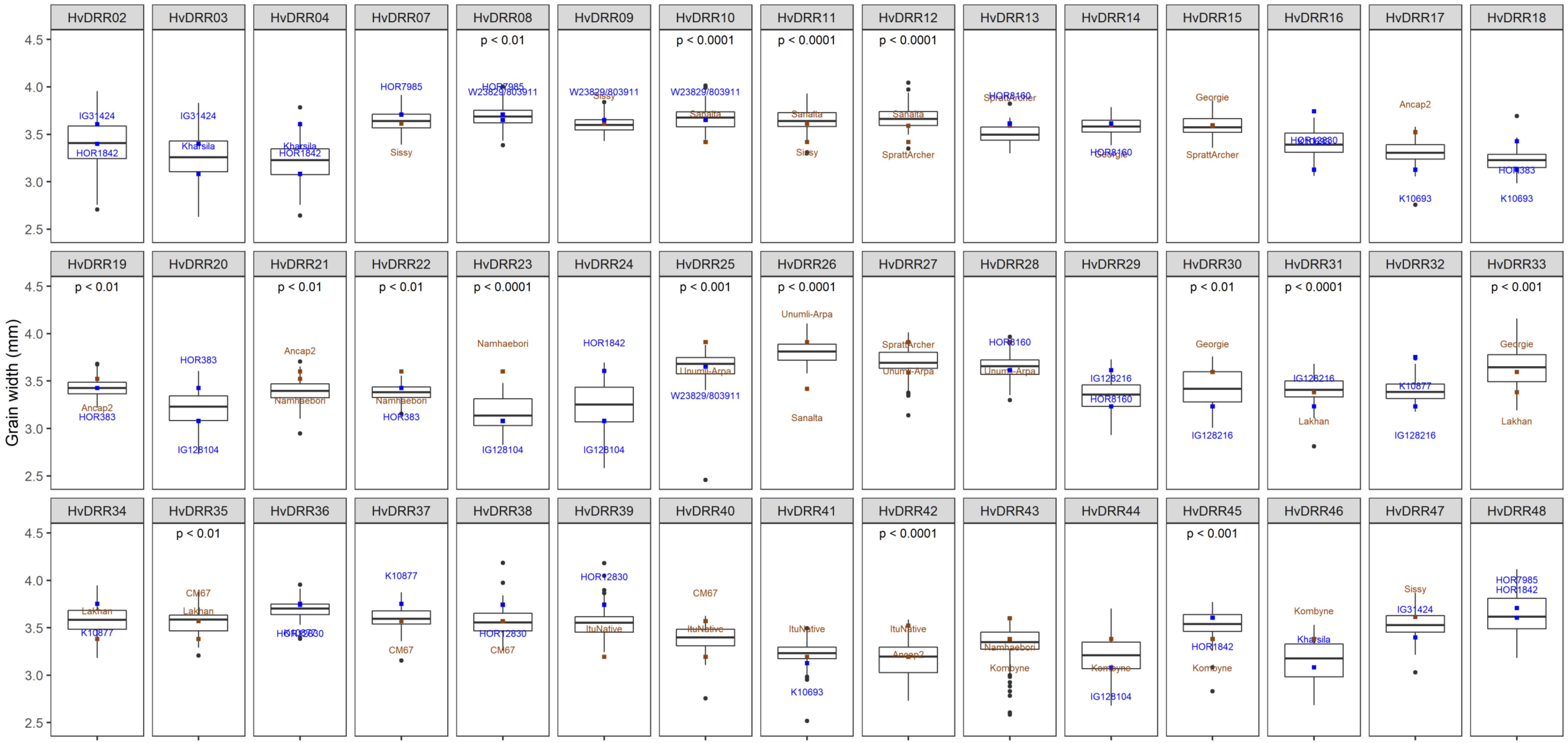

Fig. S4. Boxplot of adjusted entry means of the recombinant inbred lines of the HvDRR populations for grain width in mm. The square dots indicate the adjusted entry mean of the parental inbreds of the respective recombinant inbred line populations. Brown and blue colors designate germplasm-type cultivars and landraces, respectively. The p-value above the boxplot indicates a significant mean differences between the segregating populations and the respective parental inbreds.

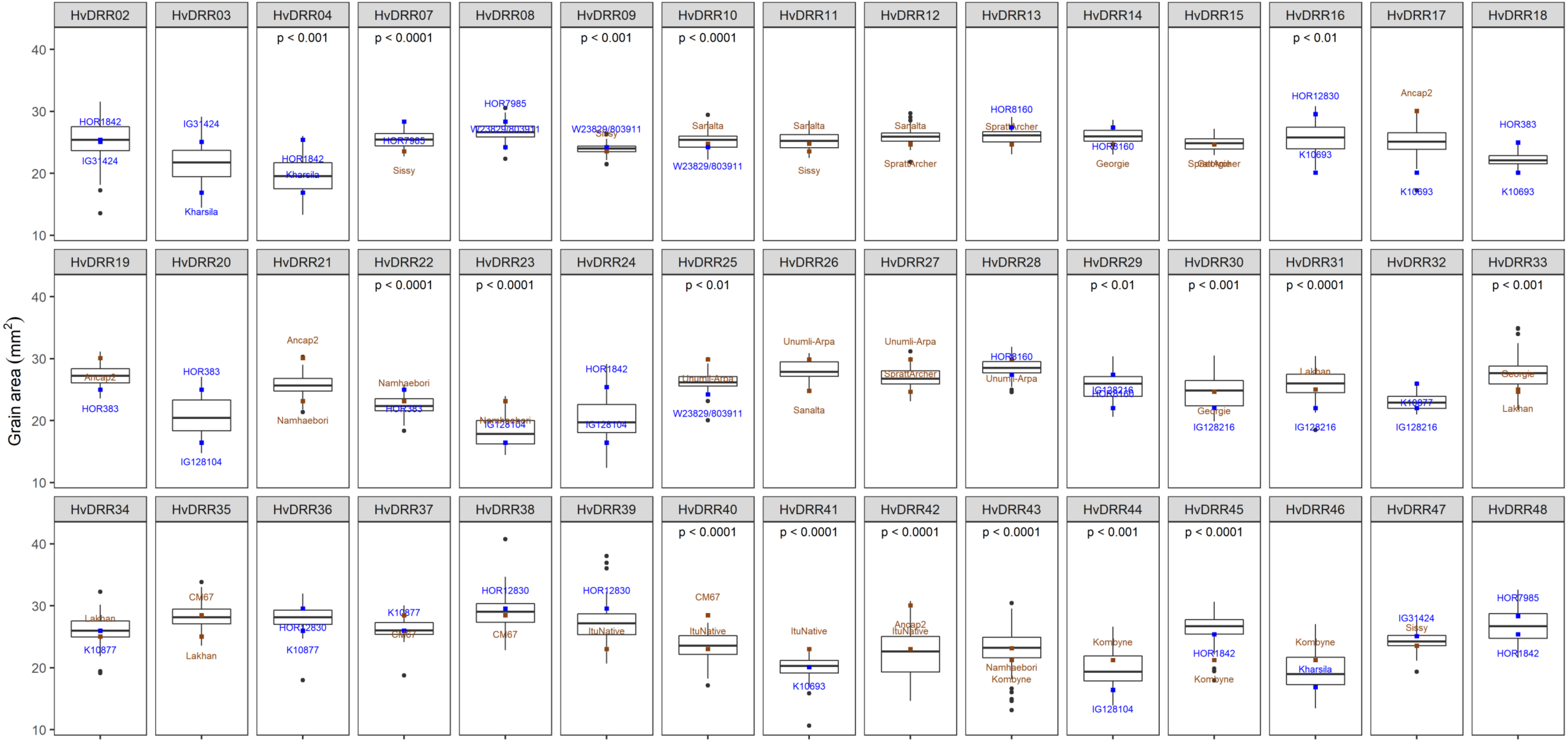

Fig. S5. Boxplot of adjusted entry means of the recombinant inbred lines of the HvDRR populations for grain area in mm². The square dots indicate the adjusted entry mean of the parental inbreds of the respective recombinant inbred line populations. Brown and blue colors designate germplasm-type cultivars and landraces, respectively. The p-value above the boxplot indicates a significant mean differences between the segregating populations and the respective parental inbreds.

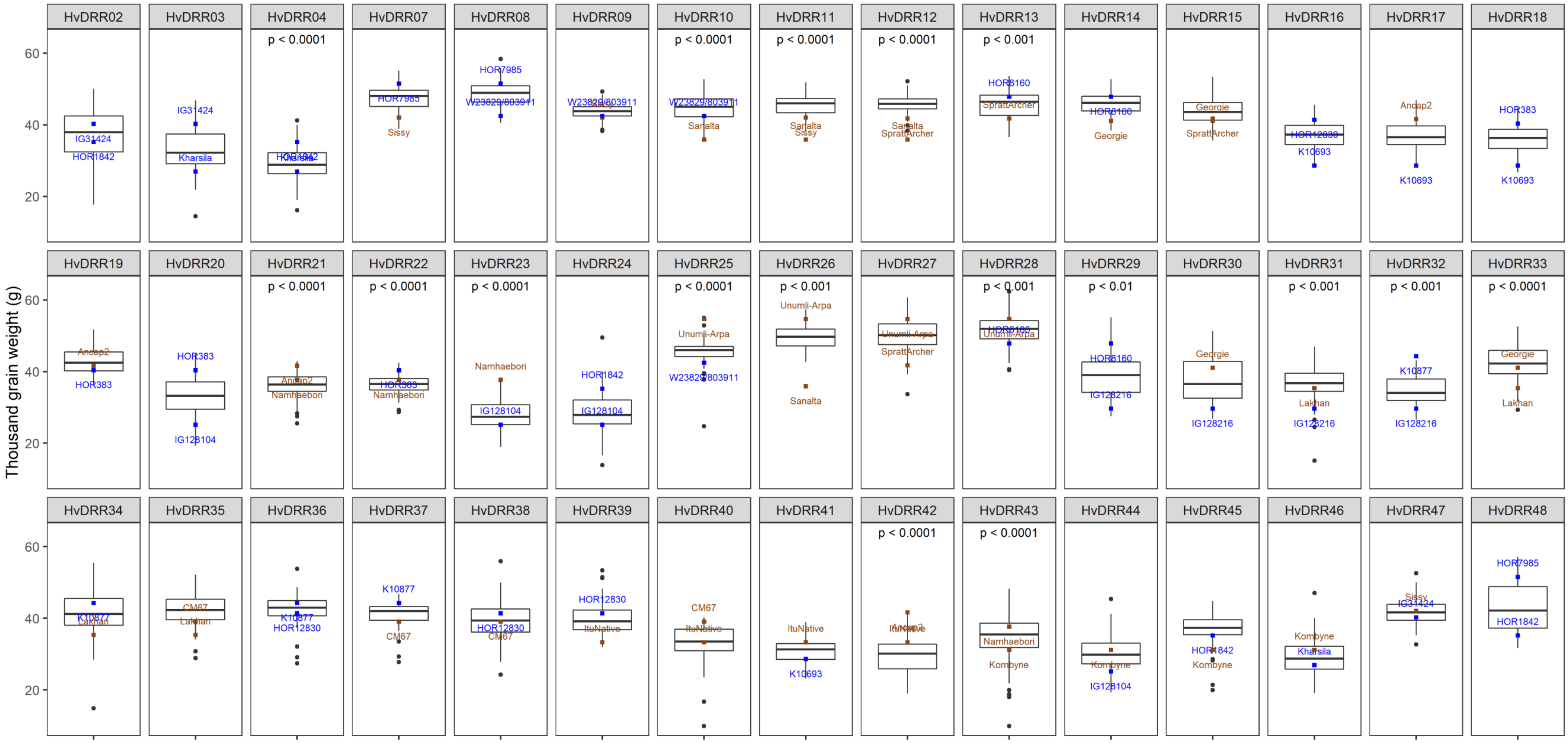

Fig. S6. Boxplot of adjusted entry means of the recombinant inbred lines of the HvDRR populations for thousand-grain weight in grams. The square dots indicate the adjusted entry mean of the parental inbreds of the respective recombinant inbred line populations. Brown and blue colors designate germplasm-type cultivars and landraces, respectively. The p-value above the boxplot indicates a significant mean differences between the segregating populations and the respective parental inbreds.

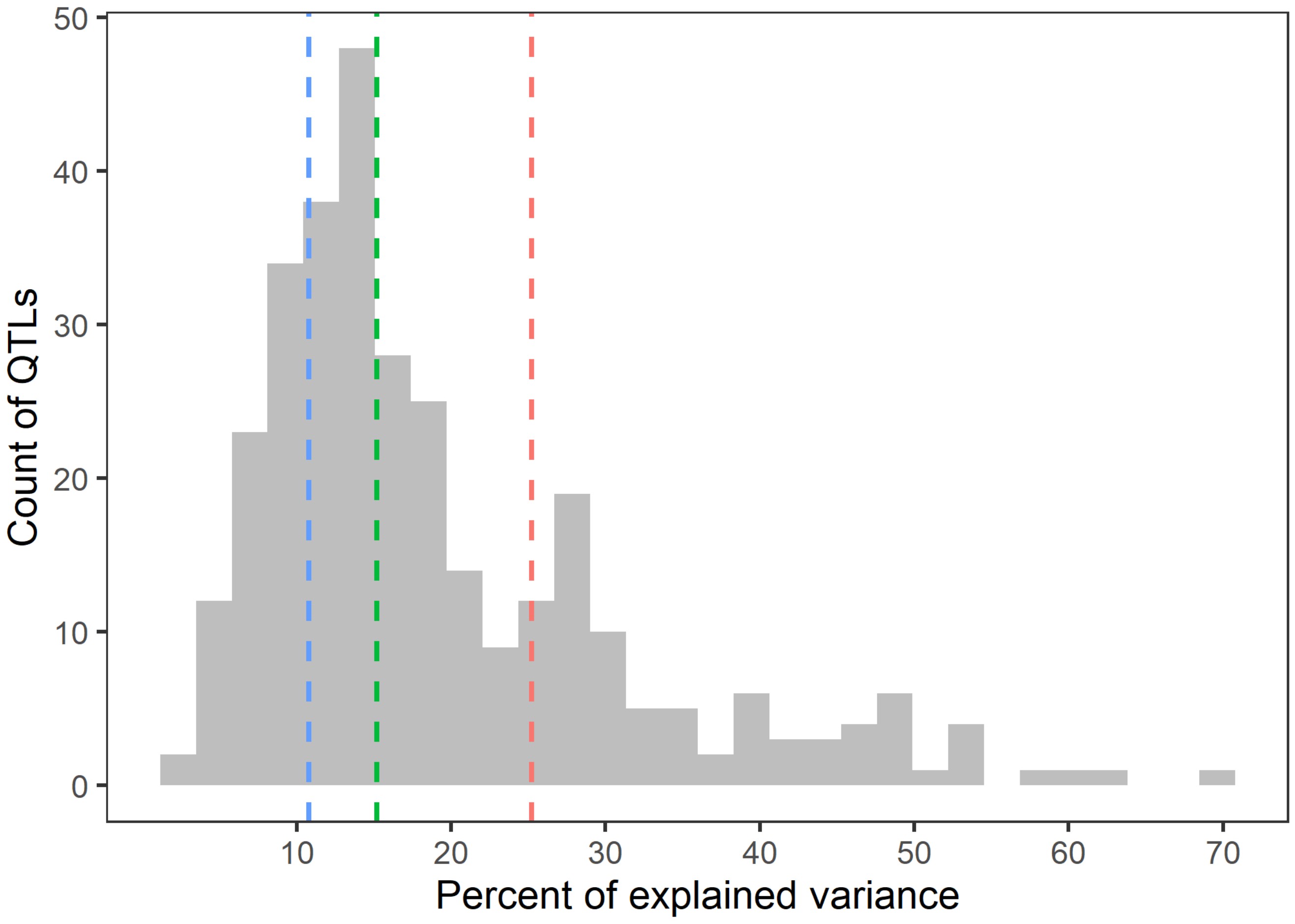

Fig. S7. Distribution of the percentage of variance explained by quantitative trait loci detected in single population analyses for grain size and thousand-grain weight in 45 HvDRR sub-populations. The blue, green, and red dotted lines indicate the first quartile, median and third quartile of the distribution.

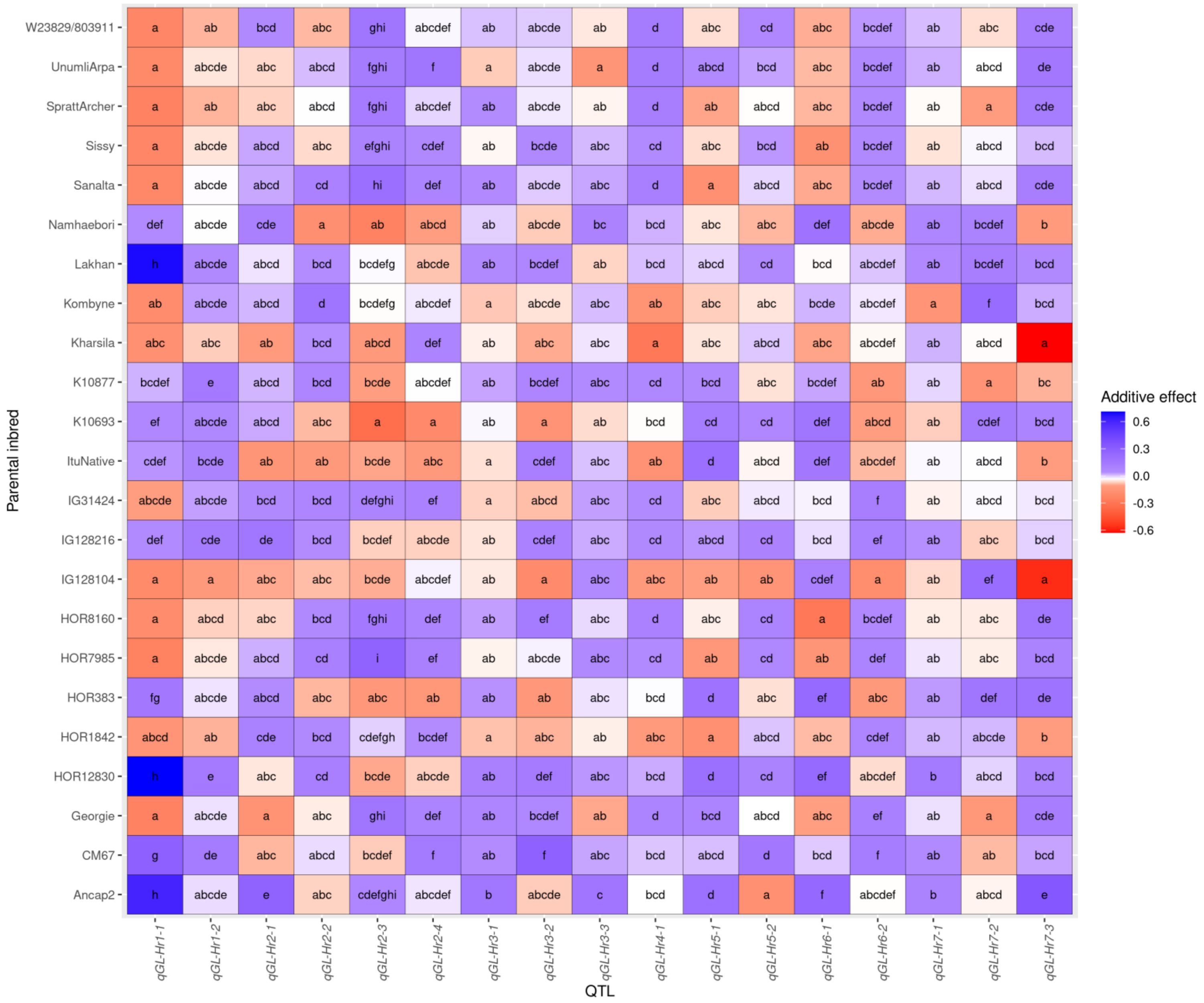

Fig. S8. Multiple comparisons of the standardized allele effect for grain length quantitative trait loci (QTLs) detected in a multi-parent population analysis using a parental model. The standardized allele effect for an inbred is the difference between the mean of the estimated additive effect for 23 inbreds and the estimated allele effect of the corresponding inbred. The color code indicates the magnitude of the standardized allele effect. Indexed letters indicate the significant difference ($p \leq 0.05$) between the genotypes not sharing the same letter by Tukey's HSD test.

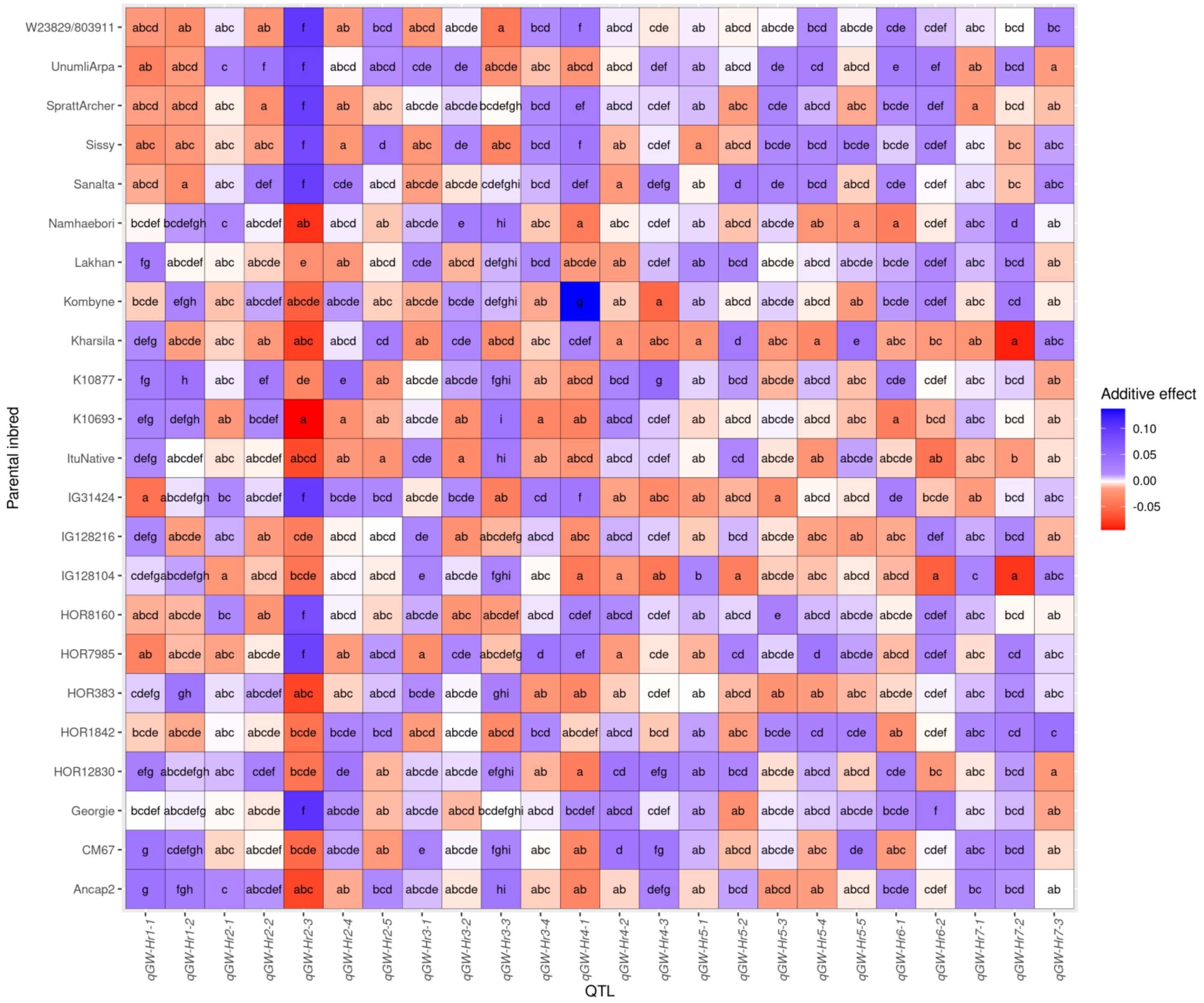

Fig. S9. Multiple comparisons of the standardized allele effect for grain width quantitative trait loci (QTLs) detected in a multi-parent population analysis using a parental model. The standardized allele effect for an inbred is the difference between the mean of the estimated allele effect for 23 inbreds and the estimated additive effect of the corresponding inbred. The color code indicates the magnitude of the standardized allele effect. Indexed letters indicate the significant difference ($p \leq 0.05$) between the genotypes not sharing the same letter by Tukey's HSD test.

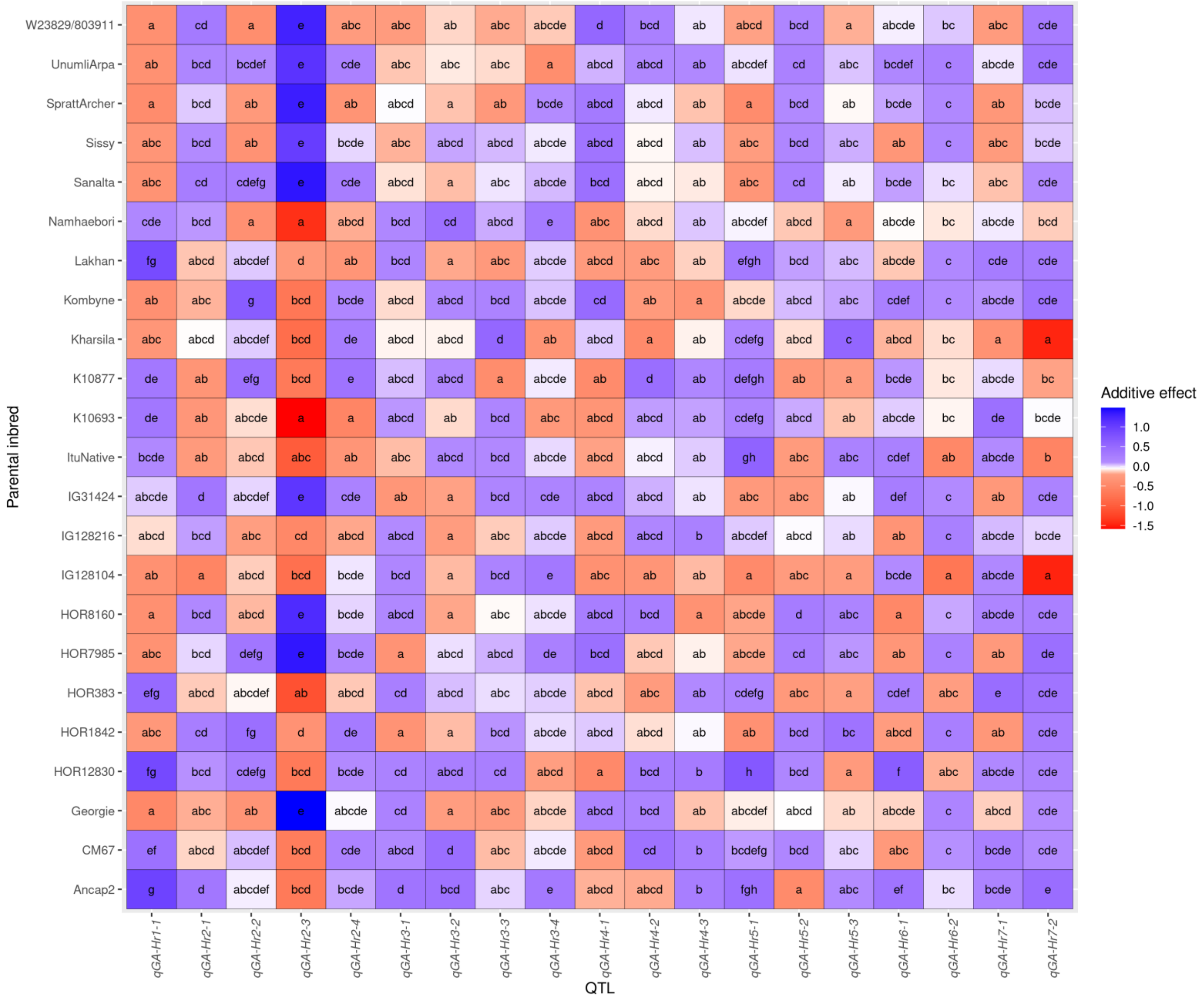

Fig. S10. Multiple comparisons of the standardized allele effect for grain area quantitative trait loci (QTLs) detected in a multi-parent population analysis using a parental model. The standardized allele effect for an inbred is the difference between the mean of the estimated allele effect for 23 inbreds and the estimated additive effect of the corresponding inbred. The color code indicates the magnitude of the standardized allele effect. Indexed letters indicate the significant difference (p ≤ 0.05) between the genotypes not sharing the same letter by Tukey’s HSD test.

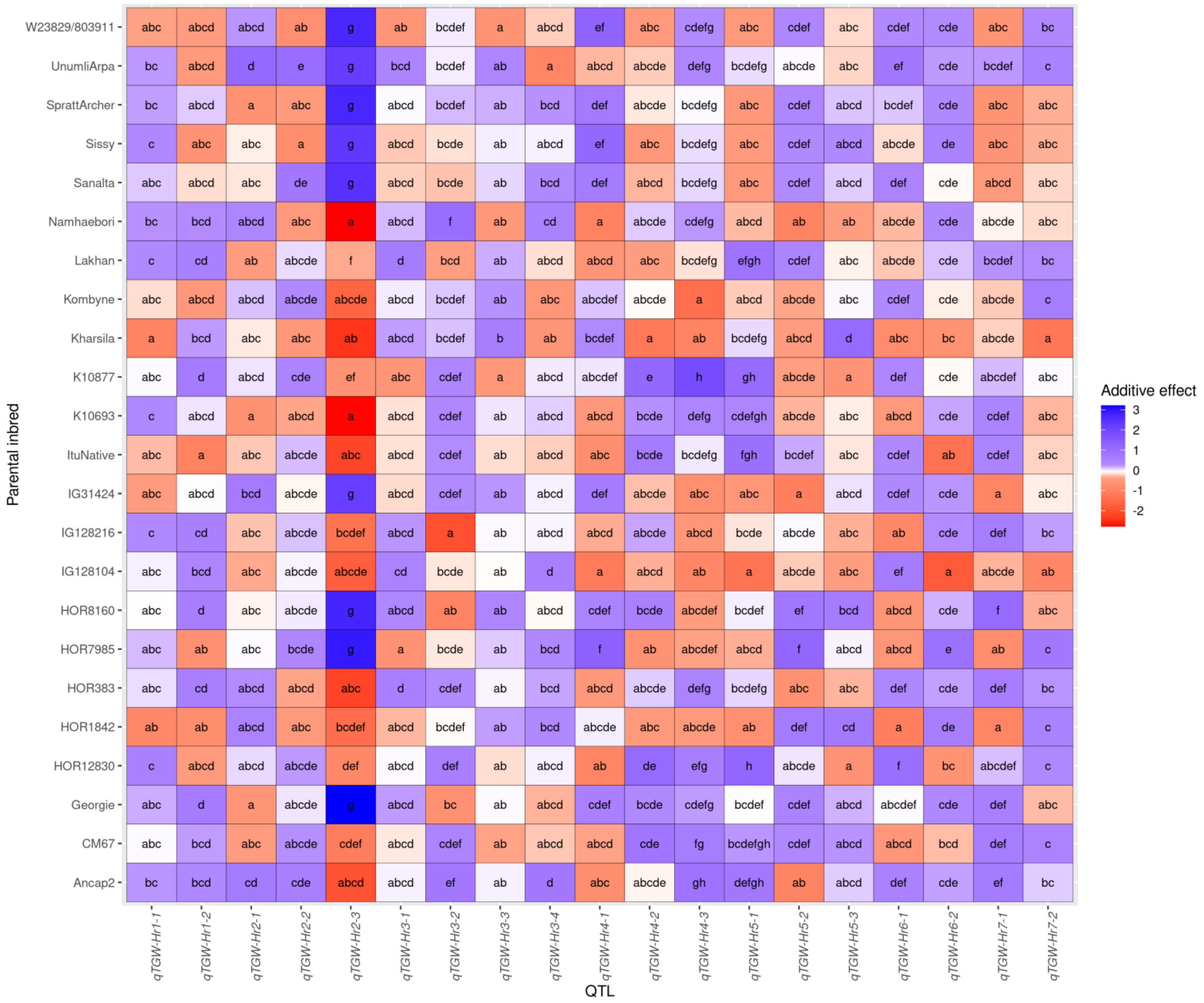

Fig. S11. Multiple comparisons of the standardized allele effect for thousand grain weight quantitative trait loci (QTLs) detected in a multi-parent population analysis using a parental model. The standardized allele effect for an inbred is the difference between the mean of the estimated allele effect for 23 inbreds and the estimated additive effect of the corresponding inbred. The color code indicates the magnitude of the standardized allele effect. Indexed letters indicate the significant difference ($p \leq 0.05$) between the genotypes not sharing the same letter by Tukey's HSD test.

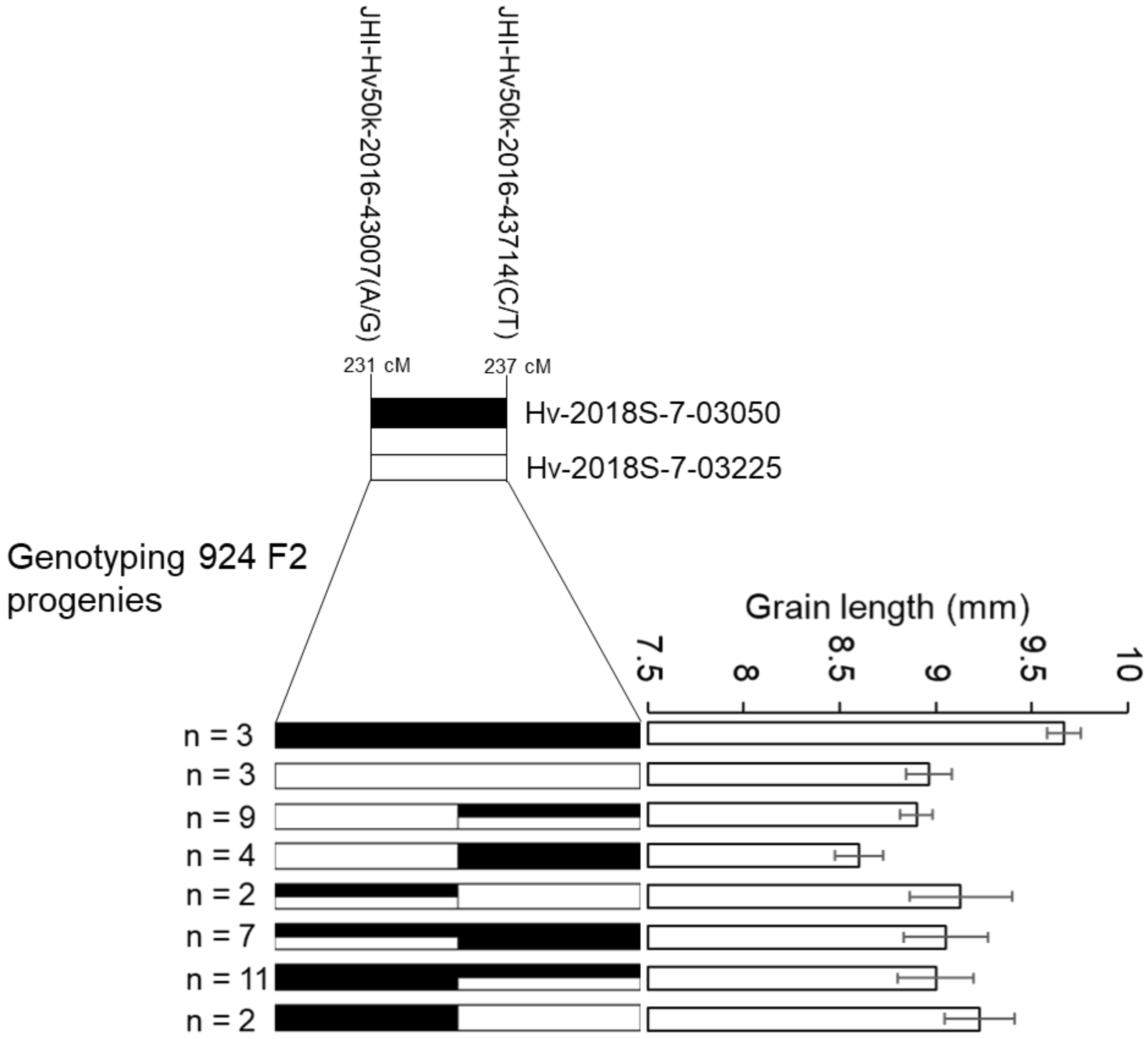

Fig. S12. QTL allele effect validation at *qHvDRR-GS-6* on chromosome 1. Two recombinant inbred lines of the HvDRR33 sub-population bearing separate QTL alleles at *qHvDRR-GS-6* but being monomorphic for the other sub-population specific QTLs were selected to produce a high-resolution segregating population. Recombinant F2 progenies segregating for *qHvDRR-GS-6* were selected by genotyping at the left and right border of the QTL. The grain length of the recombinant F2 plants and selected non-recombinants from the segregating population was evaluated. The black and white bar represents the allele from Lakhan and Georgie, respectively.

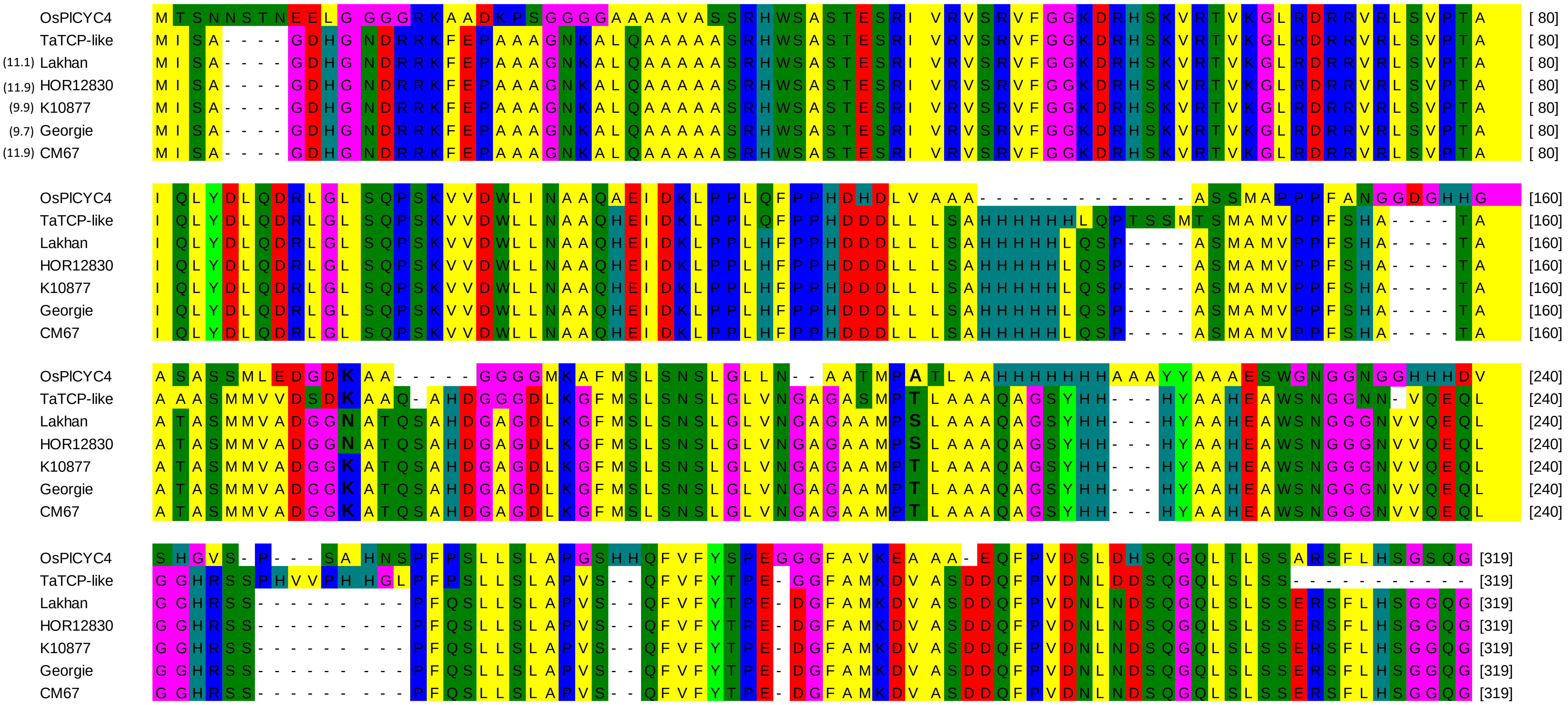

Fig. S13. Protein alignment of a candidate gene underlying *qHvDRR-GS-6* on chromosome 1. SNPs caused amino acid substitutions in the conserved domain of HORVU.MOREX.r3.1HG0077830. The protein sequence of the barley gene was highly conserved in rice and wheat. Lakhan and HOR12830 allele at the QTL locus contributed to longer grains. Sites in bold letters indicate amino acid substitutions between parental groups with contrasting additive effects at the *qHvDRR-GS-6*.

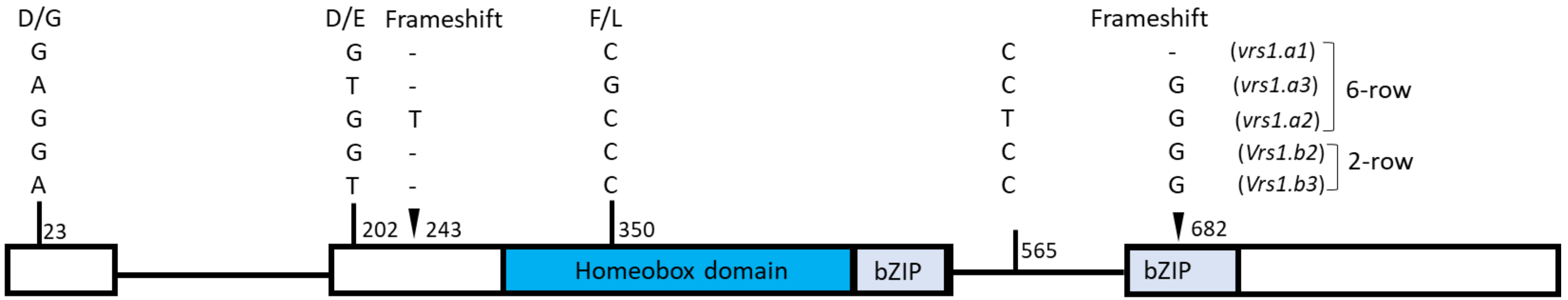

Fig. S14. The allelic variant for *Vrs1*. A major QTL at *Vrs1* locus on chromosome 2 associated with grain size and weight (*qHvDRR-GS-14*) was detected in HvDRR populations developed from the genetic cross between 2 and 6-row inbreds. The position adjacent to the polymorphic sites is the relative position from the start of the gene.
